# Development of sensor system and data analytic framework for non-invasive blood glucose prediction

**DOI:** 10.1038/s41598-024-59744-7

**Published:** 2024-04-22

**Authors:** S. V. K. R. Rajeswari, P. Vijayakumar

**Affiliations:** Department of Electronics and Communication Engineering, SRM Institute of Science and Technology, Kattankulathur, 603203 India

**Keywords:** Biological techniques, Health care, Medical research, Engineering, Biomedical engineering, Electrical and electronic engineering

## Abstract

Periodic quantification of blood glucose levels is performed using painful, invasive methods. The proposed work presents the development of a noninvasive glucose-monitoring device with two sensors, i.e., finger and wrist bands. The sensor system was designed with a near-infrared (NIR) wavelength of 940 nm emitter and a 900–1700 nm detector. This study included 101 diabetic and non-diabetic volunteers. The obtained dataset was subjected to pre-processing, exploratory data analysis (EDA), data visualization, and integration methods. Ambiguities such as the effects of skin color, ambient light, and finger pressure on the sensor were overcome in the proposed ‘niGLUC-2.0v’. niGLUC-2.0v was validated with performance metrics where accuracy of 99.02%, mean absolute error (MAE) of 0.15, mean square error (MSE) of 0.22 for finger, and accuracy of 99.96%, MAE of 0.06, MSE of 0.006 for wrist prototype with ridge regression (RR) were achieved. Bland–Altman analysis was performed, where 98% of the data points were within ± 1.96 standard deviation (SD), 100% were under zone A of the Clarke Error Grid (CEG), and statistical analysis showed *p* < 0.05 on evaluated accuracy. Thus, niGLUC-2.0v is suitable in the medical and personal care fields for continuous real-time blood glucose monitoring.

## Introduction

Invasive methods for measuring blood glucose include pricking the skin, which causes pain, anxiety, and panic. Pathology laboratory reports and additional supplies for home monitoring kits are never-ending expenses for monitoring blood glucose. Therefore, invasive methods cannot be used for continuous monitoring of blood glucose levels. Few studies have explored minimally invasive techniques for the continuous monitoring of blood glucose, where sensors are implanted in the epidermal layer of the skin to monitor blood glucose levels^1,2^. Minimally invasive techniques have not been successful because of mild irritation, discomfort, bleeding, and withdrawal from the test^3–6^.

Proof-of-concept studies were conducted using microwave sensors. In one study, a resonant sensor with an operating frequency of 9 GHz and a quality factor (Qu) of 240 was proposed to measure the dielectric constant that changes with glucose concentration, which was validated on four volunteers by comparing noninvasive and invasive blood samples^7^. Similarly, a label-free meandered sensor was implemented on an RO4003 substrate with an operating frequency of 6.21 GHz and a Qu factor of 506 to measure glucose from a glucose aqueous solution, where a sensitivity of 0.64% was obtained^8^. An inverted microstrip sensor with two metalized solutions i.e., copper and silver is proposed with operating frequencies 4.50 and 4.62 GHz with Qu factor of 16.36 and 22.0 where 93.11% data points of copper tape sensor fell in zone A and Zone B whereas 6.89% data points fall in zone D of CEG which is clinically unacceptable whereas for the silver tape sensor, 85.19% fall in zones A & B and 3.70% in zone C and 11.11% in zone D which is clinically unacceptable and thus cannot be implemented in real time^9^. An adjustable resonant frequency (F_r_)-based multiparallel complementary split-ring resonator (MP-CSRR) sensor was proposed in a recent study. After loading the glucose solution, F_r_ of 2.5–3.5 GHz is achieved, where the experimental results prove that as the glucose concentration increases, F_r_ also increases with a sensitivity of 9.59 × 10^–2^ MHz/(mg/dL), and $\left| {S_{21} } \right|$ sensitivity of 7.47 × 10^–3^ dB/(mg/dL)^67^. Upper band F_r_ between 5.42–5.87 GHz for measuring glucose concentration and lower band F_r_ between 2.40–2.48 GHz is designed for wireless communication applications where the glucose concentration was measured on a diabetic patient before and after 30 min of having food. Linear regression analysis showed that as the glucose concentration increased, F_r_ also increased^10^. In a similar work, a F_r_ with two frequencies i.e., at 5.5 GHz and 8.5 GHz with a Qu factor of 180 and 106 is proposed where the sensor is tested on 11 volunteers. An error rate of 13% was obtained between invasive and non-invasive blood glucose measurements, which is clinically unacceptable^11^. In a parallel study, a compact branch line coupler and split ring resonator with a sensor size of 3.5 × 3.5 × 0.16 cm^3^ was calibrated with five different aqueous glucose concentrations. Sensitivity of 0.72 MHz/mg/dL^−1^ and a relative error ranging from 1 to 6.6% was achieved^12^. In a relative work, an electromagnetic coupling-based tag sensor and the reader were designed, where frequency and amplitude shifts were measured by changes in glucose concentrations of 0 and 200 mM/l solutions. An accuracy of ~ 1 mM/l and 38 kHz of resonant frequency shift was achieved^13^. A sensor with coupling between the underlying split ring resonator and loading patch with resonant frequency of 5.65 GHz and 4.34 GHz is proposed which is tested on four volunteers for 3 h per day in a total of 8 days. An R^2^ value of 0.913 between the invasive and noninvasive measurements was achieved using linear regression. Long Short-Term Memory (LSTM) was applied to predict the threshold of glucose levels after 30 min with a lookahead of 360, which is high for clinical applications^14^.

However, the studies discussed are not executed in real time^11,15^, and a few of them are executed on blood samples^8,10,12,13,15^, which is a major limitation. Executing studies on real-time blood samples and comparing them with non-invasive sensors will allow us to understand whether any interference from biological differences in blood, susceptibility to interference from fats and proteins, and physiological conditions such as breathing, sweating, cardiac activity, and dehydration can alter the measurements due to changes in the permittivity and affect the sensitivity of the sensor^13,16,17^. Studies carried out on real-time blood samples lacked sample sizes^7,10,11^, which limits the observation of different volunteer demographics/selection criteria, standard error analysis^10,11,14^ for performance, validation of sensors, and clinically unacceptable ranges of CEG^9^, and require a 15 min resting time, normal temperature, and blood pressure before measurement^7^. However, the results with good sensitivity from the above studies open the door for miniaturized sensors for wearables and are trusted options for diabetes management.

Excellent research has been conducted in the areas of thermal, electric/electromagnetic, and optical methods for noninvasive glucose monitoring, as discussed in I1, Supplementary Material 1. Among all the existing methodologies, notably microwave and NIR devices, NIR is chosen in the proposed work because of its strong penetration; non-interference of biological differences in blood, fats, and proteins; non-destructive technique; no risk of infection; safety on human skin; low cost; sample can be analyzed on the spot time without time-consuming laboratory analysis; does not induce auto-fluorescence in cells; and requires no sample preparation or manipulation with hazardous chemicals, solvents, or reagents^17,18^.

Revolutionizing medical diagnoses and NIR-based commercial glucometers are available on the market. A hybrid glucometer was released using CNOGA, with an accuracy of 95%. The device's total dimensions, i.e., 43.2 mm × 47.65 mm × 74 mm, weighing 99.9 gms, make it non-portable. The cost of the device is $420. The device is minimally invasive and requires frequent changes in strips, thereby adding additional costs to the device^19^. Another drawback is the accuracy, where the Mean Absolute Relative Difference (MARD) was 18.1%, 91.1% of data points fell in zone A, and 7.8% in zone B of the Clarke error grid (CEG) analysis^20^. Similarly, the glucometer by Helo Extense World Global Network takes into account external factors such as temperature variability, and the effect of pressure and sweat to measure blood glucose and has not been approved by the FDA^21,22^. Likewise, the glucometer by Wizmi, WEAR2B Ltd is under clinical trial, where a study obtained 93% in zone A and 7% in zone B in CEG^22,23^. A discussion of commercial devices in areas other than optical methods is presented in I2, Supplementary Material 1.

Considering the challenges of pricking the finger, allergies, inaccuracies, high errors, unreliability, frequent changing of sensors, non-portability, and cost of the existing market solutions, there is a need to implement potent NIR technology with the integration of cutting-edge technologies i.e., Artificial Intelligence (AI) and Data Science for accurate predictions, lesser error, reliability and low cost for more sophisticated and efficient non-invasive diabetic management solutions.

The current state of the art in noninvasive blood glucose measurements in IR and NIR regions is thoroughly discussed. Prototypes from the literature are presented in T1 Supplementary Material 2.

A recent study proposed an Internet of Medical Things (IoMT) based wearable device. It integrates a photoplethysmography (PPG) device with 950 nm and 650 nm. A light-weight 1-dimensional input-reinforced deep neural network is employed, where an MAE of 23 mg/dL and Mean Absolute Percentage Error (MAPE) of 17.8% with 100% predictions falling under A and B of the CEG at the testing phase is achieved^24^. The model was not trained above 200 mg/dL which may lead to bias during real-time measurements and has a high MAE for clinical acceptance which is a limitation of the study.

In a parallel study, an LED of 940 nm was employed where the systolic and diastolic peaks were acquired from the PPG signal. A Standard Percentage Error (SEP) of 2.1835 was achieved^25^. There is a need to remove the first five seconds of the signal to avoid motion artifacts, which implies a limitation, along with an absence of analytical standards and evaluations that can validate the proposed sensor for real-world deployment of the device.

In a similar study, the Perfusion Index (PI) was obtained from PPG signals. The sensor was developed by combining an LED with a wavelength 940 nm with an ultrasonic sensor with an operating frequency of 40kHz. The finger was tied with a band to the sensor to avoid motion artifacts. Pearson’s coefficient of r =  − 0.90, *p *< 0.001, and MAE of *p *< 0.0051 were achieved between the predicted and reference blood glucose levels^26^. The major limitation of the study is that approximately 77.78% of participants were aged between 18 and 25 years, which may generalize the results. Unusual PI is uncommon at this age limit, which may alter the result if 25-and above-aged participants are tested with the model. The model can be trained using ML algorithms by integrating data science to handle participant demographics and PI.

A low-cost infrared (IR)-based 940 nm emitter was employed. The number of components involved in designing the working model was $18.A fuzzy logic algorithm was employed which achieved an error of prediction between ±5% and ±10%, which is clinically high, which is a limitation^27^. A wide range of patient demographics were not considered in this study, which may led to bias. The model is trained on blood glucose levels between 103 and 175 mg/dL, whereas it is recommended that the model must be trained with higher glucose levels for calibration and sensitivity. Various standards and evaluations were performed to validate the device.

A multisensory system was developed by employing an NIR wavelength of 1370 nm and 1640 nm and radiofrequency sensor between 36.50 and 41.50 GHz. A random forest (RF) algorithm was employed to achieve a Root Mean Square Error (RMSE) of 21.06 mg/dL, Mean Absolute Relative Difference (MARD) of 7.31%, and 96% under clinically acceptable zones A and B in the CEG^28^. The performance of the device is a limitation of this study, which can be improved in terms of accuracy and reduction in error for real-world deployment of the sensor. The model can be trained by considering fasting, postprandial, and random blood glucose samples collected from diabetic and non-diabetic volunteers to observe the performance of the model.

A multiple photonic-band near-infrared (mbNIR) sensor with a Shallow Dense Neural Network (SDNN) was proposed. A sensor with six 850 nm emitters and detectors was employed, which achieved an accuracy of 97.8% with a precision of 96.0%, sensitivity of 94.8%, and specificity of 98.7%. The detection limit of the proposed device was 60–400 mg/dL with a prediction error of ± 15^29^. Although the error is limited by the International Standard ISO, it can still be reduced and the accuracy can be improved for precise prediction during the practical deployment of the sensor.

In a similar study on detecting hemoglobin (Hb), blood glucose, and Creatinine (Cr), a PPG signal was acquired from a fingertip video. A source and detector at 850 nm for Hb, 950 nm for blood glucose, and 1150 nm for Cr were employed. A Deep Neural Network (DNN) is applied, which achieved an accuracy of 90.2% for blood glucose, 92.2% for hemoglobin, and 96.9% for creatinine^30^. The process of detecting blood glucose is a major limitation as it is non-portable, and deploying the application on different mobile phones can lead to errors in readings owing to different camera resolutions.

In an experimental trial, reflection spectroscopy between 1100 nm and 1825 nm was employed, where an SEP of 36.6 mg/dL and MARD of 23% were achieved, which is a limitation of the study for practical deployment^31^. Moreover, noninvasive measurements are taken from the lip, which can lead to infection if not sterilized properly and cannot be made as a portable device.

A parallel study employed two 940 nm LED sources, one 940 nm LED detector, and two 1300 nm detectors. Multiple polynomial regression (MPR) was applied with an Average Error and MARD of 6.09 % for capillary and 4.88 % and 4.86 % for serum glucose, respectively^32^. The size of the prototype can be reduced, which is a limitation of this study, along with a reduction in error for accurate prediction.

A sensor size of 15 × 15 mm^2^ wearable band-type system was developed by employing four emitter LEDs with wavelengths of 950 nm, 850 nm, 660 nm, and 530 nm and a transmitter of 400–1100 nm. Pulsatile signals were recorded to avoid a high SNR and baseline wander, in the resting position. The average correlation coefficient $R_{p}$ of 0.86, and SPE of 6.16 mg/dL were obtained, which is a limitation of the study for practical deployment of the sensor. The reliability of the device was tested by comparing the heartbeat between PPG and Electrocardiography (ECG) signals, and by investigating changes in blood glucose levels in a day^33^.

The research gaps identified in the literature are addressed as follows.

- Non-portable^25–32^ and wearable devices^24,33^ did not focus on ambiguities such as skin color variation, ambient light, pressure of the finger on the sensor, and reliability, making the device unsuitable for continuous monitoring of blood glucose accurately.
- The proposed methodologies have a high MAE/MARD/prediction error, which makes the device non-replicable using invasive or minimally invasive methods.
- The devices have been tested on normal patients^25,28,30,33^, non-diabetic patients with chronic health disorders^24,29^, and a few diabetic patients^26,27,31,32^.
- The cost of the developed prototypes in the existing literature is estimated from a minimum of $100 to $300, which is not suitable for continuous monitoring of blood glucose, is non-portable, and is non-reliable with a higher error in predictions.

An accurate, portable, and low-cost sensor system is needed to handle ambiguities such as skin color variation, ambient light, and pressure on the sensor for predicting blood glucose levels. Extending the existing literature and overcoming the challenges of commercial devices, our proposed work expands the existing methods and commercially available devices by developing a reliable prototype integrating Artificial Intelligence (AI) and Data Science to develop a data analytic framework. The proposed work was designed to handle skin color variation, the presence of ambient light, and pressure on the sensor. Machine Learning (ML) models are applied to validate the developed framework that achieved maximum accuracy compared to the existing literature. A high degree of accuracy implies its application in better diabetic management at a low cost. The ease of use of this prototype is an additional advantage.

The contributions of this proposed study are as follows.

- Development of a low-cost ($87.37) NIR spectroscopy-based noninvasive portable finger and wrist sensor prototype to detect blood glucose levels continuously.
- A novel data analytic framework was designed to improve the accuracy from 71% for niGLUC 1.0v (first version) to 99.96% for the proposed device, niGLUC 2.0v.
- The accuracy of the developed sensor system was tested for its reliability and stability in the presence of skin color variations, ambient light, and finger pressure.

The rest of the paper is presented in the following consequential manner: Sect. 2 presents the methods for selecting measurement sites, the principle of blood glucose measurement, the hardware architecture of niGLUC-2.0v, cost comparison of niGLUC-2.0v with commercial devices, development of a data analytic framework for niGLUC-2.0v, overcoming the challenge of skin pigmentation, predictive analysis, experimental design, data collection, testing the accuracy of niGLUC-2.0v for variation in ambient light, and handling other ambiguities. The results and discussion are presented in Sect. 3, where predictive analysis, validation of niGLUC-2.0v, Bland–Altman plot, CEG analysis, statistical analysis, and comparison with recent research works are covered. The paper ends with Sect. 4, an exposition of the conclusion.

## Methods

The current section elaborates on the selection of measurement sites, the principle behind light absorption, and the hardware architecture. An adjustment factor was proposed to handle skin color variation. The adjustment factor was evaluated for different intervals and multiplied by the voltages generated from niGLUC-2.0v. The dataset was fed into the data analytic framework, where EDA was applied and achieved the highest accuracy. The proposed data analytic framework improves the accuracy of the developed device in the presence of ambiguities. A cost comparison of niGLUC-2.0v was performed using commercially available devices. ML algorithms and metrics evaluation are presented to validate the developed device. The experimental procedure and data collection are elaborated with the hardware setup.

### Selection of measurement sites

Noninvasive blood glucose measurements from the lips, cheeks, tongue, eyes, earlobe, fingertip, and wrist have been reported in the literature^34–40^. The fingertip and wrist have thin skin folds and are a source of blood vessels i.e., capillaries where blood glucose can be easily found, which lie much above the fat layer of the skin. NIR requires thin skin folds and has the property of transilluminance i.e., when NIR light passes through skin and tissues, it penetrates the underlying structures such as blood vessels where absorption takes place. Information-rich spectral intervals are found in the first overtone and combination-band vibrations^31,41,42^. Based on the biophysical properties, better absorption properties of NIR, and to avoid the risk of infection, measurement alterations and, ease of handling the device, fingertip, and wrist were chosen in the proposed work.

### Principle of blood glucose measurement-Absorption physics at Near-Infrared region

**Figure 1:**
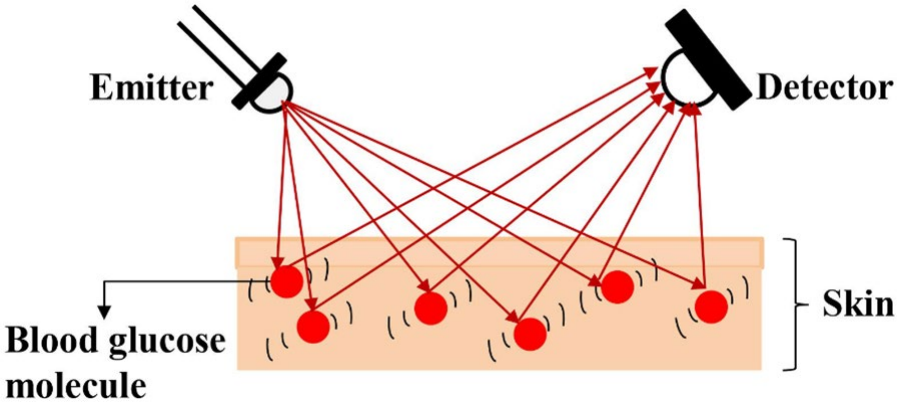
Absorption and reflection of light by glucose molecule by Beer Lambert’s law.

The absorption of light by the blood glucose molecules (C_6_H_12_O_6_) is due to the overtone and combination bands, which cause photons to absorb and induce molecular vibrations. These vibrations are due to covalent bonds, which behave like springs through bending and stretching. Stretching of the CH and OH bonds was observed in this region. Molecules vibrate and absorb when the frequency of light matches the vibrating frequency^43–45^. This absorption is described by the Beer-Lambert law, as illustrated in Fig. 1.

According to the Beer-Lambert law, the absorbance of any solution is proportional to its concentration and the path length traveled by light rays^46^. When the blood glucose concentration is high, the absorbance of photons by blood glucose molecules is high with decreased scattering and a shorter optical path^47^. The principle of blood glucose measurement is written as,

1 $$R = R_{0} e^{{ - \mu_{eff} l}}$$

where R = is the reflected light intensity, R_0_ = is the incident light intensity, l = is the length of the optical path inside the tissue, and (${\upmu }_{{{\text{eff}}}}$) = is the effective attenuation coefficient with respect to the absorption and reduced scattering coefficients. The effective attenuation coefficient is written as,

2 $$\left( {\mu effl} \right) = \sqrt {3\gamma a\left( {\gamma a + \gamma s^{\prime}} \right)}$$

3 $$\gamma a = 2.303\varepsilon C$$

4 $$\gamma s^{\prime} = \gamma s\left( {1 - a} \right)$$

where ε = molar extinction coefficient, C = tissue chromophore concentration, μ_s_^′^ = reduced scattering coefficient, a = average of the cosine of the scattering angles.

From Eq. 1, it can be inferred that the glucose molecules absorb the light produced by the NIR emitter, and the reflected light is measured at the detector as voltage. The absorption, scattering, and transmission of light through the sample depended on glucose concentration.

### Rationale behind selection of sensor

The sensor was selected on the basis of the wavelength and penetration depth of the skin. Capillary loops consisting of blood glucose molecules are present in the dermal layer of the skin at a 2.0 mm depth, which is easily penetrated by NIR sensors^48^. From the literature, glucose absorption peaks are found at 660 nm, 940 nm, 1550 nm, and 1650 nm, where the penetration depth of light is highest at 940 nm^28,32,49,50^. Below 700 nm and above 950 nm, the penetration of light is challenging owing to the strong absorption from hemoglobin and water molecules. The penetration depth increases to 900–1000 nm and then decreases^48–51^. At 940 nm, attenuation by other constituents of the blood, such as water, hemoglobin, and melanin, is minimum^52–54^. Therefore, 940 nm was selected for this study.

### Hardware architecture of niGLUC-2.0v

**Figure 2:**
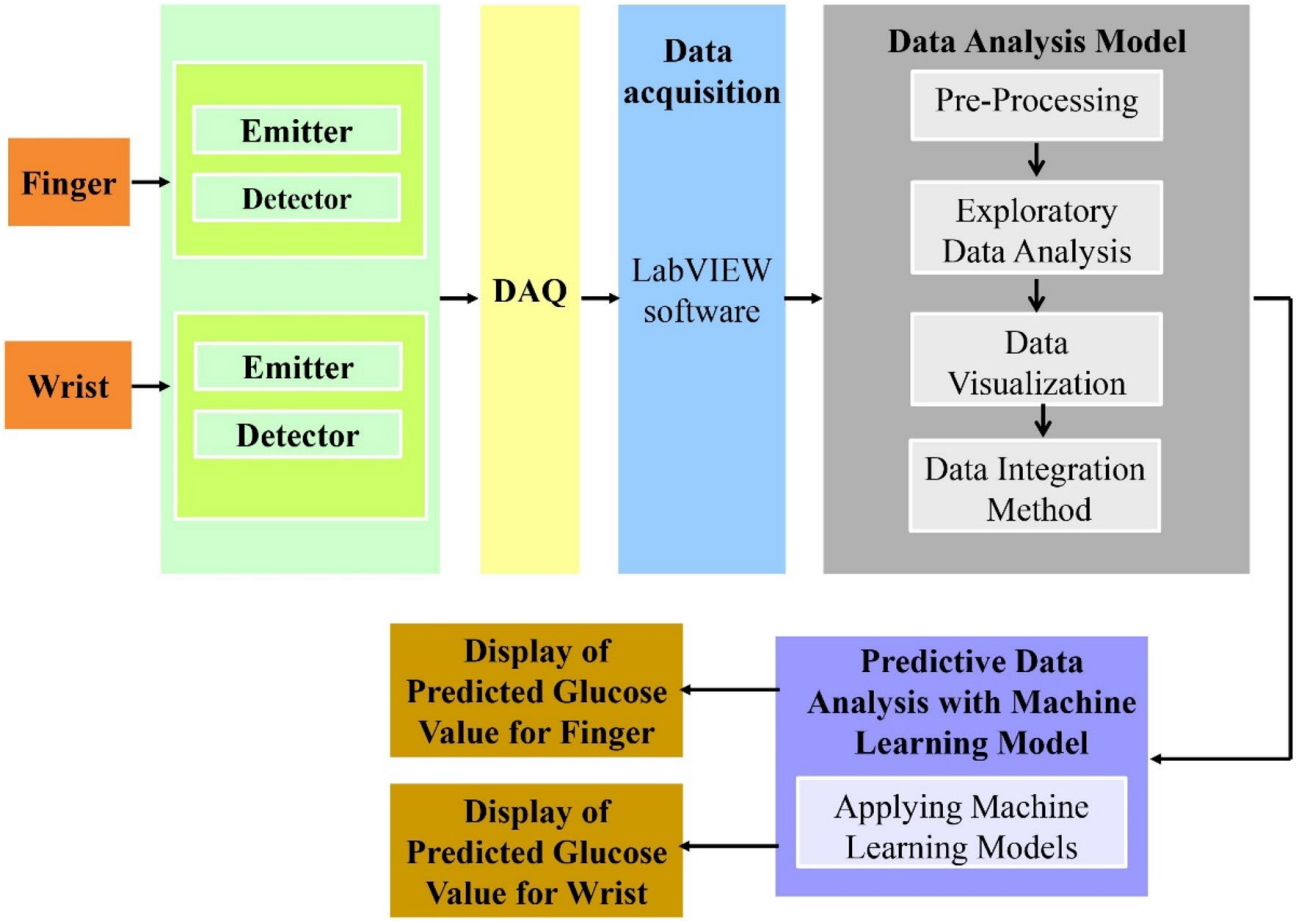
Block diagram of the proposed sensor system.

The hardware design was conceptualized using SW-NIR between 700 and 1300 nm. NIR sensor with an emitter of wavelength 940 nm and a 900–1700 nm detector were chosen to detect the blood glucose molecule. The sensitivity of the sensor is 0.9–0.95 amperes/watt. The specificity of the sensor was 0.5 amperes/watt. The range of sensor to detect blood glucose levels is in between 0 to 0.3 mm. Two sites were selected for the detection of blood glucose levels: the finger and the wrist. A block diagram of the prototype is shown in Fig. 2. Aluminum gallium arsenide (GaAlAs) LEDs were chosen because the p-surface was coated with silicon nitrate (SiN_4_O_12_), which helped to reduce the interference of ambient light and provided better stability at the output. Half angle of the LED was 40°. The LEDs were placed on the same side so that the reflected light was captured at the detector, and thus, a 180° phase shift occurs between them. The distance between the emitter and the detector was 5.5 mm. The niGLUC-2.0v operates with a 5 V power supply and 2 A current. The operating power of the finger sensor is 0.5 W and 0.7 W for the wrist sensor. The current consumed by the finger and wrist sensor was 100 mA. When light from the NIR emitter passes through the blood, the detector detects the reflected light from the blood in the form of a signal^55^. The amplitude of the signal depends on the blood glucose concentration. If the blood glucose concentration is high, the reflected signal is low and vice versa.

**Figure 3:**
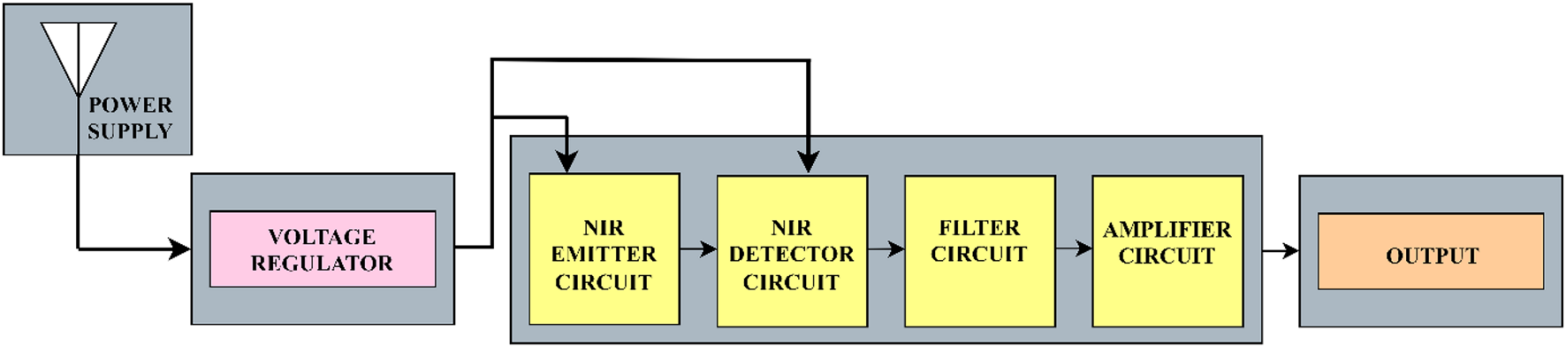
Block diagram of circuit protection and components.

A block diagram of the circuit protection and its components is presented in Fig. 3.Various circuit protection mechanisms have been implemented^56,57^. However, in the proposed work, as the circuit works on a 5 V power supply (small voltage application), a voltage regulator is implemented to protect the circuit from overvoltage conditions, voltage spike suppression, and thermal protection. The internal circuitry was protected by resistors and the ground to protect the components. The sensor consists of an emitter and a detector circuit. The reflected signal at the detector was passed through a low-pass filter and then amplified using a power amplifier. The output consists of an amplified analog signal fed to the DAQ. Radiation safety is considered based on the Incoherent Visible and Infrared radiation on Non-Ionizing Radiation Protection (ICNIRP) guidelines that state thermal injury of the cornea in case of direct eye exposure > 1000 s^58^.Nevertheless, in the proposed study, there was no direct eye contact with the sensor, and the exposure to radiation was < 60 s.The components were secured in a Printed Circuit Board (PCB) covered with a black body and a transparent window underneath that allow light to pass from the emitter into the skin. Data acquisition (DAQ) is employed to convert the analog signal into a digital signal, where the voltage values can be viewed using LabVIEW software. The output voltages were received serially in frames.100 frames were collected as one corresponding sample. Data analysis is carried out in the sequence of pre-processing of the data and applying a data analytic framework, i.e., employing EDA, data visualization, data integration, and predictive analysis. Predictive analysis was performed by applying ML models in which the blood glucose levels were predicted.

### Cost comparison of niGLUC-2.0v with commercial devices

The cost of the proposed niGLUC 2.0v device is listed in Table 1. Cost comparisons were performed between invasive, minimally invasive, implantable, and niGLUC-2.0v. It can be inferred from the table that the total cost of pathology lab reports, including Fasting Plasma Glucose (FPG), postprandial (PP), and Glycated Hemoglobin (HbA1C) profiles calculated four times a year at Apollo Hospital, is estimated to be $35^59^. An Accu-check glucometer costs $430, including additional supplies (glucometer, lancet, and strip of 50 counts) purchased four times a year^60^. The total cost of a minimally invasive device for one Dexcom G6 transmitter and three sensors was $1060. The sensor and transmitter must be changed for 10 and 90 days^61^. Eversense E3 is an implantable device available with insurance, which costs $675.3 for one-time insertion and sensor removal^62^. In the proposed study, LabVIEW was implemented for the convenience of data collection. Arduino-based open software will be used at final product. niGLUC-2.0v costs $85.6 as a one-time purchase, a non-invasive and portable device that does not change the sensors or transmitters.

**Table 1:** Cost comparison of invasive, minimally invasive, implantable, and niGLUC-2.0v.

Method of testing	Device/source	Price ($)
Invasive	Apollo	37.57
	Accu-chek glucometer	428.60
Minimally invasive	Dexcom G6	1056.89
Implantable	Eversense E3	673.18
Proposed device (ni-GLUC-2.0v)	Finger & wrist prototype	87.35

### Development of data analytic framework for niGLUC-2.0v

**Figure 4:**
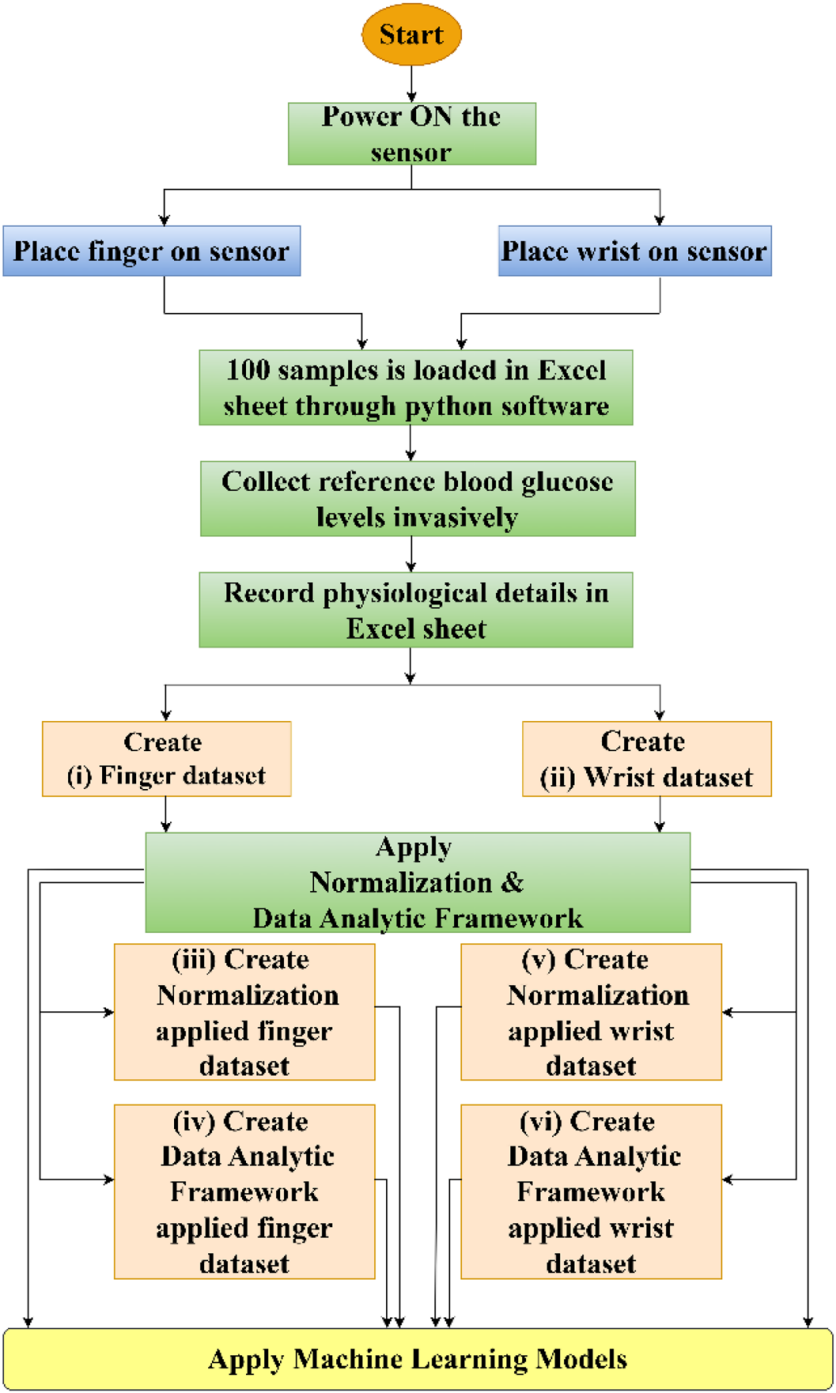
Flow chart of the proposed work.

The flowchart of the proposed work is shown in Fig. 4. The finger and wrist sensors were switched on, and the values were recorded from the sensors. One hundred samples from the sensor were recorded and saved in an Excel file along with physiological details of the patient. A normalization and data analytic framework was applied to the datasets on which ML algorithms were used to accurately predict blood glucose levels.

### Overcoming the challenge of skin pigmentation

**Figure 5:**
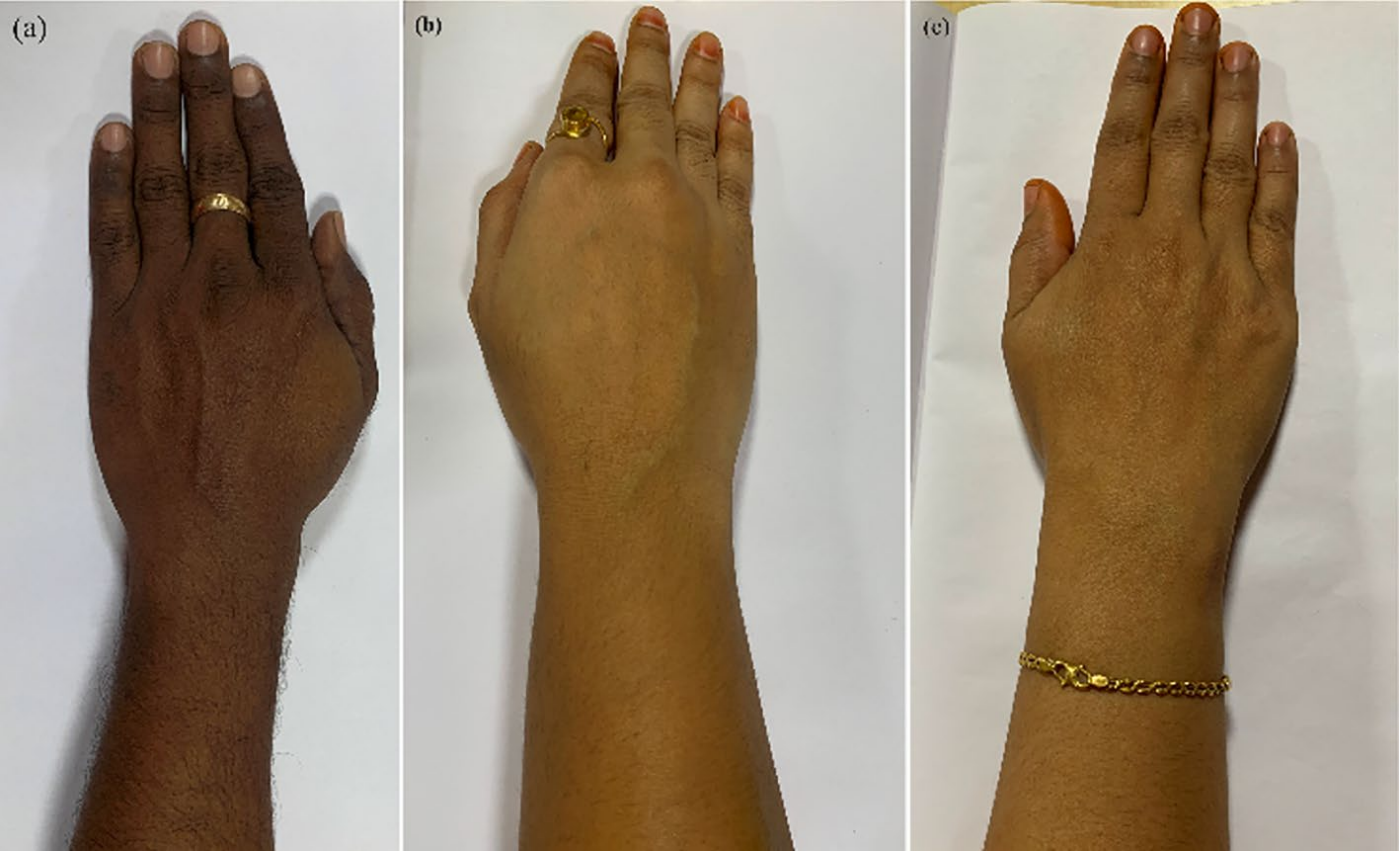
Three skin color variations: (**a**) Dark skin tone; (**b**) Wheatish skin tone;(**c**)Fair skin tone.

Three healthy volunteers age group-33–35 with dark, wheatish, and fair skin tones were selected as shown in Fig. 5a–c. Volunteers were asked to fast the previous night. The same quantity of breakfast and lunch were consumed. Fasting and postprandial blood glucose values were recorded invasively using a home monitoring kit and non-invasively using niGLUC-2.0v, as illustrated in Table 2. A total of 44 readings were collected over five days. For comparison, blood glucose levels can be divided into a range of 5, i.e., between 91–95 mg/dL, 121–125 mg/dL, 126–130 mg/dL, and 131–135 mg/dL. It can be observed from Table 6 that the postprandial values (after breakfast) obtained invasively for volunteers 1 and 3 were the same, i.e., 128 mg/dL. In contrast, a difference can be noted in the corresponding voltages, i.e., 0.15548 V for volunteer 1 and 0.164059 V for volunteer 2. A variance of 0.008579 V was observed for the wrist. A significant difference can be observed between volunteer 2 with 0.055806 V at 126 mg/dL when compared with volunteers 1 and volunteer 3, with the blood glucose level falling within the same interval, i.e., 128 mg/dL. A difference of 0.099674 V was observed. Although the differences among the three healthy volunteers were negligible skin color was a significant factor of interference when a large dataset was considered with different age groups, sex, BMI, and other physiological factors. There is a need for NIR sensors that consider skin color interference for accurate blood glucose predictions.

**Table 2:** Blood glucose levels and corresponding voltages in different skin tones.

Vol	BF/L	Invasive (mg/dL)	Wrist (V)	Skin color
1	BF	128	0.164059	Dark
	L	122	0.051148	Dark
2	BF	133	0.142742	Wheatish
	L	126	0.055806	Wheatish
3	BF	128	0.15548	Fair
	L	95	0.002872	Fair

Vol: volunteer; BF: breakfast; V: voltage;

The current work proposes a novel interval-based adjustment factor, as detailed in Algorithm 1 in Table 3, for handling skin color pigmentation. The dataset was arranged in ascending order of the reference blood glucose values. The dataset was divided into dark, wheat, and fair skin colors. The invasive blood glucose values from all skin colors and their respective niGLUC-2.0v values were grouped at an interval of 5. The variance of the three skin tones falling within the same interval was calculated using Eq. (2). The variance was evaluated to be 0.000375 for $G_{3}$ (T). The correction factor with respect to variance was evaluated at each interval, as mentioned in Eq. (3). A correction factor of 0.019366 was obtained for $G_{3}$(T). The adjustment factor was calculated from the invasive blood glucose level and correction factor mentioned in Eq. (4). It was calculated for all voltages falling within the interval. Similarly, the adjustment factor was calculated for all intervals. An adjustment model was created and fed into the data analytic framework to predict blood glucose levels.

5 $${\text{X}}_{{{\text{var}}}} = \frac{{\sum \left( {{\mathbf{C}}_{{\mathbf{i}}} - {{\varvec{\upmu}}}} \right)}}{{\left( {{\text{n}} - 1} \right)}}$$

6 $${\text{F}}_{{{\text{correct}}}} = \frac{1}{{\surd {\text{X}}_{{{\text{var}}}} }}$$

7 $${\text{A}}_{{{\text{factor}}}} = {\text{X}}_{{{\text{var}}}} \times {\text{F}}_{{{\text{correct}}}}$$

where ${\varvec{C}}_{{\varvec{i}}}$** = **reference values, µ = mean of 3 skin tones, n = total number of measurements.

**Table 3:**
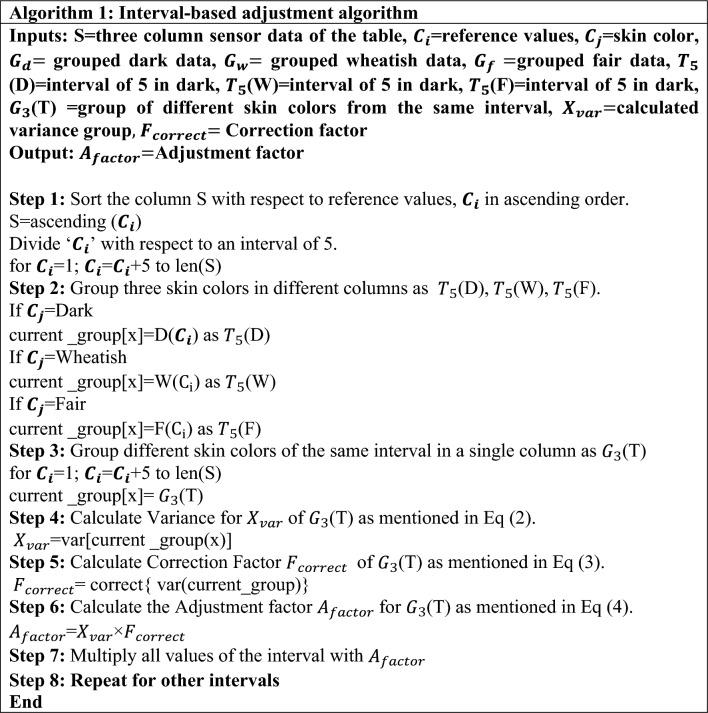
Interval-based adjustment algorithm.

#### Exploratory data analysis for niGLUC-2.0 V

EDA is implemented to understand the pattern in data visualization to rectify errors, anomalies, and outliers that may take place during data collection.

It is used to obtain the desired level of prediction by estimating the parameters and margins of error from existing data^63^. A data integration method was employed to preprocess the dataset. The output of Algorithm 1 is the adjustment factor at different intervals multiplied by the blood glucose levels. The dataset with a multiplied adjustment factor of voltages from niGLUC-2.0v was fed into the data analytic framework for accurate blood glucose prediction.

The step-by-step flow of the data integration method is presented in Algorithm 2 in Table 4. The dataset was arranged in ascending order of the reference blood glucose levels obtained invasively. The reference blood glucose levels were divided into intervals of 5, i.e., from 81–85 mg/dL to 486–500 mg/dL. For ease of calculation, the reference blood glucose values in (mg/dL) were converted into millimoles (mmol). The EDA was applied to the dataset. At this step, the reference blood glucose values within an interval of five were averaged. The dataset was updated by assigning the averaged reference values to the respective hardware values in the interval. For example, for a blood glucose interval of (4.22–4.44) mmol, the average value is 4.33 mmol, as illustrated in Table 4. The average blood glucose value was assigned to the hardware values of the blood glucose level. Every hardware-generated value was assigned to the averaged reference value within that particular range. The process was repeated at five intervals for invasively obtained blood glucose levels. A new dataset was created with a column of average blood glucose values, where predictive analysis was applied, as discussed in the next section.

**Table 4:**
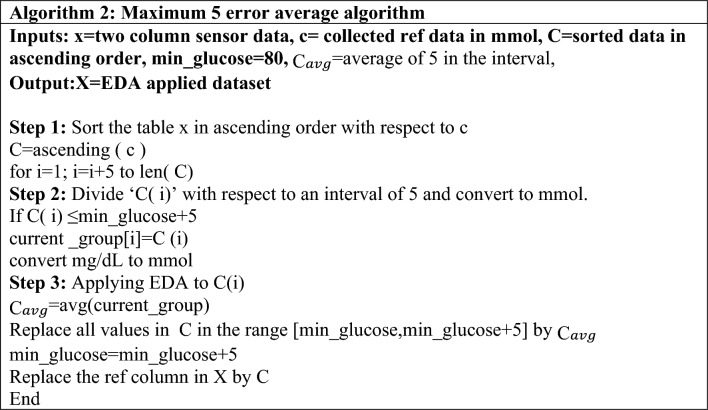
Exploratory data analysis on the dataset by setting a threshold for distribution.

### Predictive analysis

This section presents an exploration of different ML algorithms and evaluation metrics to analyze the performance of the sensor system.

#### Choosing the algorithms

The proposed work consists of a dataset with continuous variables for prediction implying a regression problem. Predictive models are selected by comparing and analyzing various regression algorithms from the literature. From the profound literature that discussed on the rationale behind the selection of regression algorithms, it was reported that the performance of predictive models depends on the methodologies implemented, the dataset created^50,64–69^, the size and heterogeneity of the dataset^70^. Regression algorithms differ in their principles of operation, advantages, and limitations. In LR, the algorithm determines the best-fitting line to minimize the difference between the independent and dependent variables. Although its simplicity is advantageous, outliers, and nonlinear patterns cannot be captured which is a limitation^71,72^. PR, which is an extension to LR, models the variables as an nth-degree polynomial function to handle complex patterns; however, its major limitation is its susceptibility to overfitting at higher polynomial degrees^71,72^. However, Lasso CV which is a technique of LR was chosen because it minimizes the residual sum of squares (RSS) and adds regularization. The regularization parameter can discard important features when coefficients shrink to zero. The advantage of Lasso CV over LR and PR is its ability to automatically select features, leading to a sparse model and handling multicollinearity in the dataset^71–73^. RF works on the principle of aggregating the predictions from multiple decision trees and has the advantage of handling high-dimensional data and overfitting. The limitation is that hyperparameter tuning is required because it is less interpretable than the individual decision trees^71,73^. RR works on the principle of penalizing the squared values of regression coefficients by not shrinking the parameters exactly to zero and handling multicollinearity, which is an advantage. The limitation of this method is the inability to perform automatic feature selection and handle sparse data^72,73^. In k-NN, the dependent variable is predicted by averaging the values of the nearest neighbors to k. Although it has the advantage of simplicity and ability to handle complex patterns, it requires scaling of features, which is a limitation, thus increasing the computational overload^71^. DT operates on the principle of partitioning the tree into branches based on feature values corresponding to a decision rule. It is simple and automatically performs feature selection which is advantageous^71,73^. However, overfitting is a disadvantage. Ensemble learning models, that is bagging and boosting on k-NN and DT, have been explored in the literature. Bagging works on the principle of model learning independently of each other in parallel and aggregating to determine the model average. Boosting works on the principle of sequential and adaptive learning to improve the model prediction of the learning algorithm. The main advantage of applying the bagging algorithm to k-NN and DT is its ability to reduce variance and overfitting, but computational complexity poses a limitation. However, applying a boosting algorithm to k-NN and DT limits the bias and variance, but is prone to overfitting because of weak learners, which is a limitation^74^. In contrast, the NN model works on the principle of learning complex patterns and relationships from interconnected neurons with the advantage of high scalability and flexibility posing an advantage. The disadvantage of the NN model is the requirement of a large amount of data for training and the computational overload^71,72^. In the proposed work, LR is applied to determine the nature of the dataset, and based on its complexity of nonlinearity, PR is applied to determine the relationship between the variables. Lasso CV and RF were chosen to avoid overfitting for generalization and regularization and to improve the model performance. As the dataset was multicollinear, the RR was selected. Because the dataset was nonlinear and complex, k-NN and DT were selected. Bagging and boosting algorithms were selected to analyze the performance of the models. Therefore, in this proposed work, LR, PR, Lasso CV, RF, RR, k-NN, k-NN Bagging and k-NN Boosting, DT, DT-Bagging, DT-Boosting, and NN were applied to analyze the best algorithm for real-time collected datasets.

#### Evaluation metrics for validating niGLUC-2.0v

Evaluation metrics are required to measure and build a generalized model. In the proposed study, the MAE, MSE, and r2score were evaluated for the optimized prediction of the glucose concentration, as detailed in Eqs. (8)–(10):

8 $$MAE = \frac{1}{N}\mathop \sum \limits_{I = 1}^{N} \left| {BG_{pred} - BG_{ref} } \right|$$

9 $$MSE = \frac{1}{N}\mathop \sum \limits_{I = 1}^{N} \left| {BG_{ref} - BG_{pred} } \right|^{2}$$

10 $$r2\_score = 1 - \frac{{SS_{res} }}{{SS_{total} }}$$

11 $$SS_{res} = \mathop \sum \limits_{I} \left( {BG_{ref} - BG_{pred} } \right)^{2}$$

12 $$SS_{total} = \mathop \sum \limits_{I} \left( {BG_{ref} - \overline{{BG_{pred} }} } \right)^{2}$$

13 $$\overline{{BG_{pred} }} = \frac{1}{N}\mathop \sum \limits_{I = 1}^{N} BG_{ref}$$

## Experimental design and data collection

This section presents the experimental design and data collection procedure.

### Healthcare data standards and data characteristics

Standard healthcare precautions are taken to reduce the risk of bloodborne or pathogen transmission from recognized and unrecognized sources. Hand hygiene, respiration, and cough etiquette were strictly followed. The selection criteria of the volunteers are presented in Table 5.

**Table 5:** Selection criteria of volunteers (n = 101).

Mean age	57
SD	11
Age in range	25–78 years
Age in group	(20–35):4
	(35–50):22
	(50–65):61
	(65–90):14
Gender	M = 57, F = 44
Diabetes duration	Atleast 1 year of clinical diagnosis with diabetes
Inclusion criteria	Volunteers with proper mental health and cognition
Exclusion criteria	Volunteers who have hypoglycemic episodes with unconsciousness, seizure disorder are excluded from the study

SD: standard deviation; F: female; M: male.

The existing literature^24,29,32^ considered > 100 patients. However, the sensor was not tested above 200 mg/dL^24^, and on a similar number of volunteers^32^, whereas in^29^, the inclusion criteria were anyone > 18 years who had volunteers with chronic kidney disease, which limits the analysis to the possibility of variance and leads to bias. Studies where < 100 patients were considered included only healthy participants^28,31^, diabetic, and non-diabetic^26,27^ and few did not provide any information regarding the volunteers^25,30,33^. Volunteer demographics play a significant role in the validation and sensitivity of the sensors. It helps augment the quality of care by detecting variances in treatment and ensuring that competent care is provided^75^. A total of 101 patients were considered in the proposed study, with an age range of 25 to 78 years, with 57 male and 44 female volunteers. Volunteers with proper mental health and cognition were recruited as the inclusion criteria, whereas volunteers who had hypoglycemic episodes with unconsciousness, and seizure disorders were excluded from the study due to the possibility of measurement errors. The sensor was validated in all volunteers, where the blood glucose values ranged from 80 to 488 mg/dL, thus validating the robustness of the sensor.

The following steps taken to maintain the standard health protocol and a description of the collected data characteristics are as follows:

- Ethical clearance was obtained from SRM Medical College Hospital & Research Centre (ethical clearance number 8274/IEC/2022).
- Relevant guidelines and regulations were implemented for all methods.
- All experimental protocols were approved by the SRM Medical College Hospital and Research Center.
- A doctor from the SRM Medical College Hospital and Research Centre was involved in the current study.
- Informed Consent was obtained from each volunteer.
- Particulars of volunteers, such as, name, age, sex, details of the meal taken, the time between reading and meal were recorded, if an individual had diabetes, if the volunteer was on any medications, physical activity in daily life, height, weight, sleep status, stress, SP02, hair on the wrist and any other health complications were recorded.
- The finger of the volunteer was cleaned with isopropyl alcohol before reading the invasive sample.
- A new set of lancets and strips were used for each sample.
- The finger of the volunteer is pricked from the lancet. The drop of blood was placed on the strip, which was then inserted into the glucometer of the invasive device. The blood glucose values of the device were noted. The skin surface was cleaned again with an isopropyl alcohol solution and cotton.
- A volunteer is explained about the noninvasive method of obtaining blood glucose values from the hardware. The volunteer was asked to insert their fingers into the wearable prototype for the measurement of blood glucose values. Wrist wearables were tied to the wrist of the volunteer to obtain blood glucose values from the wrist.

**Figure 6:**
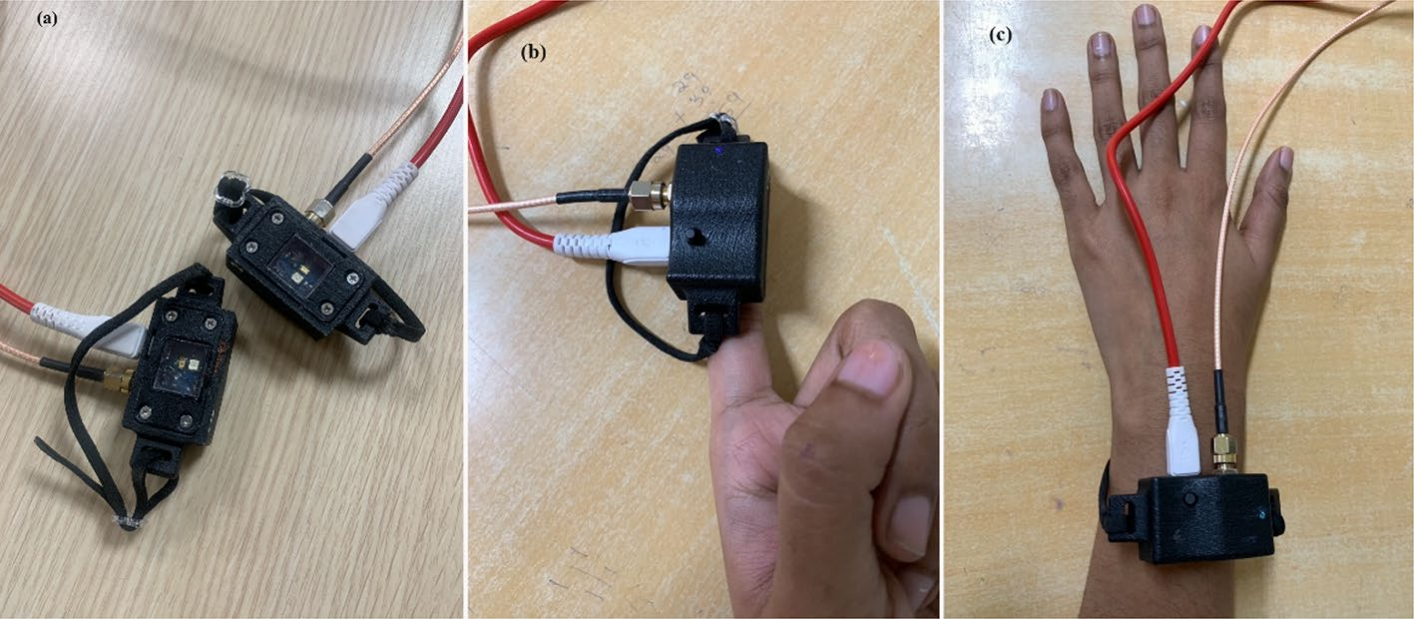
The hardware setup of the niGLUC-2.0v: (**a**) Finger and wrist sensor; (**b**) Measurements from finger sensor; (**c**) Measurements from wrist sensor.

The finger and wrist sensors of niGLUC-2.0v are shown in Fig. 6a. The finger of the volunteer was placed on the hardware, as shown in Fig. 6b. Blood glucose levels were measured invasively using the finger prick method as a reference value. Fasting, postprandial, and random blood tests were performed using a finger prick to determine real-time blood glucose values. Similarly, the wrist sensor was worn by a volunteer, as shown in Fig. 6c. The collected data were visualized using LabVIEW software. A total of 100 data points were recorded in an Excel sheet for a single sample. To validate the proposed hardware, blood glucose measurements from the designed noninvasive hardware were compared with the blood glucose values of the invasive method blood glucose values, i.e., reference values. The finger and wrist prototype of niGLUC-2.0v was tested in 101 diabetic and prediabetic individuals. Fasting, postprandial, and random blood glucose levels were collected from males and females aged 20–90 years. The baseline data collection with samples and sex distribution of the volunteers are listed in Table 6.

**Table 6:** Baseline data collection with samples and gender distribution.

niGLUC-2.0v		
Sample group	Sample data from finger	Sample data from wrist
Diabetic samples	Male (M): 54	Male (M): 54
Age in groups (years)	Female (F): 43	Female (F):43
(20–35)	M:3, F:0	M: 3, F: 0
(35–50)	M:11, F:8	M:11, F:8
(50–65)	M:34, F:27	M:34, F:27
(65–90)	M:6, F:8	M:6, F:8
Prediabetic samples	Male (M): 3	Male (M): 3
Age in groups (years)	Female (F): 1	Female (F): 1
(20–35)	M: 1, F: 0	M: 1, F: 0
(35–50)	M:2, F:1	M:2, F:1
(50–65)	M:0, F:0	M:0, F:0
(65–90)	M:0, F:0	M:0, F:0
Repeated samples	Male(M): 3	Male(M): 3
	Female (F): 1	Female(F): 1
(50–65)	M: 1, F: 1	M: 1, F: 1
(65–90)	M:2, F:0	M:2, F:0
Total samples	Male(M): 57	Male(M): 57
Age in groups(years)	Female(F): 44	Female(F): 44
Total samples	101	101

M: Male; F: Female.

#### Normalization

Normalization was applied to remove the effect of the dark current. This is detailed in Eq. (8).

- Bare LED values -LV and niGLUC-2.0v values—HV are noted.
- The dark current value DC of the photodiode was noted by switching off niGLUC-2.0v.
- Normalization is given as,
  14 $$N = \left( {\frac{HV - DC}{{LV - DC}}} \right)100$$
  where N = Normalized value, HV = niGLUC-2.0v value, DC = dark current, LV = LED value.

### Testing the accuracy of niGLUC-2.0v for variation in the ambiance light

Light sources may interfere with sensor accuracy^76^. niGLUC-2.0v was tested in volunteers with and without diabetes. Random glucose levels in both volunteers were measured invasively and non-invasively at regular intervals. As shown in Table 7, in the presence and absence of ambient light, the noninvasive blood glucose values were close to the reference blood glucose levels obtained invasively.

**Table 7:** Validation in the presence of ambient light ON and OFF condition.

Vol	Invasive (mmol)	Ambient light ON (predicted)		Ambient light OFF (predicted)	
	Reference	Finger (mmol)	Wrist (mmol)	Finger (mmol)	Wrist (mmol)
1	21.5	21.4	22.1	21.3	22.1
	20.7	20.5	20.7	20.5	20.7
	20.6	20.4	20.7	20.5	20.7
2	5.4	5.2	5.4	5.2	5.5
	5.6	5.4	5.4	5.4	5.5
	7.3	7.2	7.1	7.1	7.3

Vol: Volunteer; mmol: millimollecule.

### Handling other ambiguities

The measurements with the finger sensor and wrist sensor prototype were performed in a stable state in the sitting position of the volunteer to minimize the effect of motion artifacts. The measurements were performed with and without pressure on the finger and wrist sensor prototypes. Two datasets were created in this study. The data analytic framework was applied to the dataset, where no difference was observed between the reference and predicted glucose levels, as illustrated in Table 8. It was observed that the pressure did not influence the device because the surface of the skin had no direct contact with the sensor, was covered with a transparent surface, and was firmly packed. The reliability of the device was tested at regular intervals in different patients.

**Table 8:** Validation in the presence of pressure on the sensor.

Vol	Invasive (mmol)	With pressure (predicted)		Without pressure (predicted)	
	Reference	Finger (mmol)	Wrist (mmol)	Finger (mmol)	Wrist (mmol)
1	11.5	11.4	11.7	11.3	11.6
	25	25	25.2	24.8	25.2
	24	23.7	23.9	24	24.2
2	15.7	15.4	15.4	15.2	15.5
	9.7	9.8	9.4	9.6	9.5
	18	17.8	17.9	18.1	18.1

Vol: Volunteer; mmol: millimollecule;

## Results and discussion

The current section discusses the results and discussion of the predictive analysis, Bland Altman analysis, Clarke error grid analysis, statistical analysis and a comparison of the current work with the present literature. Regression models discussed in the Methods section were applied to niGLUC-2.0v. Ten input features, i.e., non-invasive blood glucose value, age, sex, body mass index (BMI), details of the meal taken, if an individual is diabetic, sleep status, stress, SP02, and hair on the wrist, were considered as inputs of the model.

### Predictive analysis on niGLUC-2.0v

In this section, predictive analysis is applied to niGLUC-2.0v. LR, PR, RF, Lasso CV, RR, k-NN, k-NN bagging, k-NN boosting, DT, DT-bagging, DT-boosting, and NN are applied to the computational model to obtain optimized regression method for precise measurement of predicting blood glucose. The datasets on which the ML algorithms were applied were the finger and wrist obtained dataset, normalization applied dataset, and data analytic framework applied dataset. The calibration and comparative results of all ML models are presented in Table 9. The shaded row in the table represents the performance of the respective ML models among all ML algorithms. The datasets of the finger sensor performed well with the RR. The data analytic framework applied the dataset performed with the best accuracy, achieving an MAE of 0.15, MSE of 0.2287, and r2_score of 0.9902. Similarly, the wrist sensor prototype performed well with RR, whereas on the normalization applied, the wrist sensor performed best with the Lasso CV regression model. The wrist sensor performed best on the data analytic framework applied dataset, with an MAE of 0.66, MSE of 0.006, and r2_score of 0.9996.

**Table 9:** Analysis of calibration and comparison of ML Models for niGLUC-2.0 V.

Study	Model	Validation of different models for niGLUC-2.0v		
		MAE	MSE	r2_score
Finger sensor	LR	4.516	31.50	**− **0.31
	PR	22.81	968.64	**− **39.34
	RF	3.87	21.62	0.098
	Lasso CV	3.92	21.62	0.098
	**RR**	**4.436**	**30.34**	**− 0.265**
	k-NN	3.97	22.49	0.06
	k-NN bagging	3.96	22.45	0.063
	k-NN boosting	4.87	31.016	**− **0.29
	DT	6.93	81.042	**− **2.37
	DT bagging	4.101	26.29	**− **0.09
	DT boosting	4.64	37.06	**− **0.54
	NN	8.6058	98.0432	**− **3.08
Finger sensor-normalization	LR	4.52	29.89	**− **0.11
	PR	107.68	1160.4	**− **3623.26
	RF	4.75	30.13	**− **0.12
	Lasso CV	4.60	30.86	**− **0.15
	**RR**	**4.52**	**29.92**	**− 0.11**
	k-NN	4.26	26.86	**− **0.004
	k-NN bagging	4.27	26.62	0.004
	k-NN boosting	4.33	25.98	0.028
	DT	6.20	66.34	**− **1.48
	DT bagging	4.51	30.56	**− **0.14
	DT boosting	4.84	42.23	**− **0.57
	NN	9.3862	114.8391	**− **3.29
Finger sensor-data analytic framework applied	LR	0.1552	0.2309	0.9901
	PR	2.7642	21.457	**− **0.095
	RF	1.5517	4.9564	0.7877
	Lasso CV	0.1325	0.2054	0.1077
	**RR**	**0.1536**	**0.2287**	**0.9902**
	k-NN	3.6294	20.832	0.1077
	k-NN bagging	3.5874	20.654	0.1153
	k-NN boosting	4.2207	22.636	0.0305
	DT	0.2070	0.5096	0.9781
	DT bagging	0.4610	0.5413	0.9768
	DT boosting	0.3069	0.3602	0.9845
	NN	8.02	76.06	**− **5.49
Wrist sensor	LR	3.51	18.95	0.049
	PR	17.99	745.63	**− **28.33
	**RF**	**3.742**	**18.61**	**0.066**
	Lasso CV	3.51	18.67	0.063
	RR	3.53	19.08	0.042
	k-NN	3.76	21.62	**− **0.08
	k-NN bagging	3.70	20.67	**− **0.03
	k-NN boosting	4.16	26.29	**− **0.31
	DT	3.55	19.65	0.01
	DT bagging	4.16	23.32	**− **0.16
	DT boosting	4.27	30.89	**− **0.54
	NN	8.97	100.46	**− **4.03
Wrist sensor-normalization	LR	5.19	41.64	**− **0.03
	PR	72.218	17,046.24	**− **695.12
	RF	5.13	42.14	**− **0.05
	**Lasso CV**	**4.87**	**37.82**	**0.021**
	RR	5.190	41.68	**− **0.04
	k-NN	5.01	39.22	0.021
	k-NN bagging	4.95	38.80	0.03
	k-NN boosting	5.007	36.582	0.08
	DT	6.54	82.64	**− **1.06
	DT bagging	5.60	48.60	**− **0.21
	DT boosting	5.89	62.62	**− **0.56
	Neural Network	9.3862	114.8391	**− **3.29
Wrist sensor-data analytic framework applied	LR	0.0681	0.0062	0.9996
	PR	0.6087	1.4360	0.9467
	RF	1.6412	3.5911	0.8092
	Lasso CV	0.0643	0.0059	0.0871
	**RR**	**0.0668**	**0.0060**	**0.9996**
	k-NN	3.4455	17.1884	0.0871
	k-NN bagging	3.5086	17.45	0.0731
	k-NN boosting	4.3089	24.8238	**− **0.3183
	DT	0.2508	0.18355	0.9902
	DT bagging	0.3102	0.2408	0.9872
	DT boosting	0.1857	0.11195	0.9940
	NN	10.10	126.18	**− **4.236

LR: Linear regression; PR: polynomial regression; RF: random forest; RR: ridge regression; k-NN: k-neural network; DT: decision tree; NN: neural network.

The comparison analysis of ML algorithms and the niGLUC-2.0v sensors determines the reliability of the prototype based on the following findings: (i)The data analytic framework applied dataset performed best when compared with the non-data analytic framework applied dataset for LR, PR, Lasso-CV, k-NN, k-NN bagging, DT, DT-bagging and DT-boosting ML models. (ii) It can be inferred from the comparison of sensor and ML models that the finger and wrist sensor of the niGLUC-2.0v prototype performed best with the data analytic framework. (iii) The attempt to remove the dark current through normalization was overcome by the proposed data analytic framework, which provided the best accuracy compared with the normalization-applied dataset. Therefore, the proposed data analytic framework performed best with RR regression in the presence of skin color variation, finger pressure, and ambient light.

### Ridge regression for blood glucose prediction in ni-GLUC-2.0v

RR performed best, with the highest accuracy for the developed model, as reported in Table 9. RR is a model-tuning method that is implemented to analyze multiple multicollinear regression data. Multicollinearity occurs when a high correlation exists between independent variables, thereby raising the issue of high variance. Large variances deviate from the predicted value to the reference value, thus increasing loss. RR, which is a regularization technique, was applied by adding a penalty term to the loss function. The penalty is equal to the square of the magnitude of the coefficients. The RR minimizes the error by adding a degree of bias to the regression estimates. The challenge thrown by multicollinearity is reduced by adding a shrinkage parameter $\uplambda$ .

15 $$RR = \mathop \sum \limits_{j = 1}^{n} (y_{i} - \mathop \sum \limits_{k = 1}^{m} x_{jk} \beta_{k} )^{2} +\uplambda \mathop \sum \limits_{k = 1}^{m} \beta_{k}^{2}$$

where RR = Ridge regression*,* $y_{i}$ = dependent variable. ${\text{x}}$ = independent variable, β = coefficient, $\uplambda$ = shrinkage parameter.

The RR derived in Eq. (11) has two components. The former component represents the least-square term, whereas the latter represents the penalty term added to the least-square term to attain a low variance.

### Validation of niGLUC-2.0v

**Figure 7:**
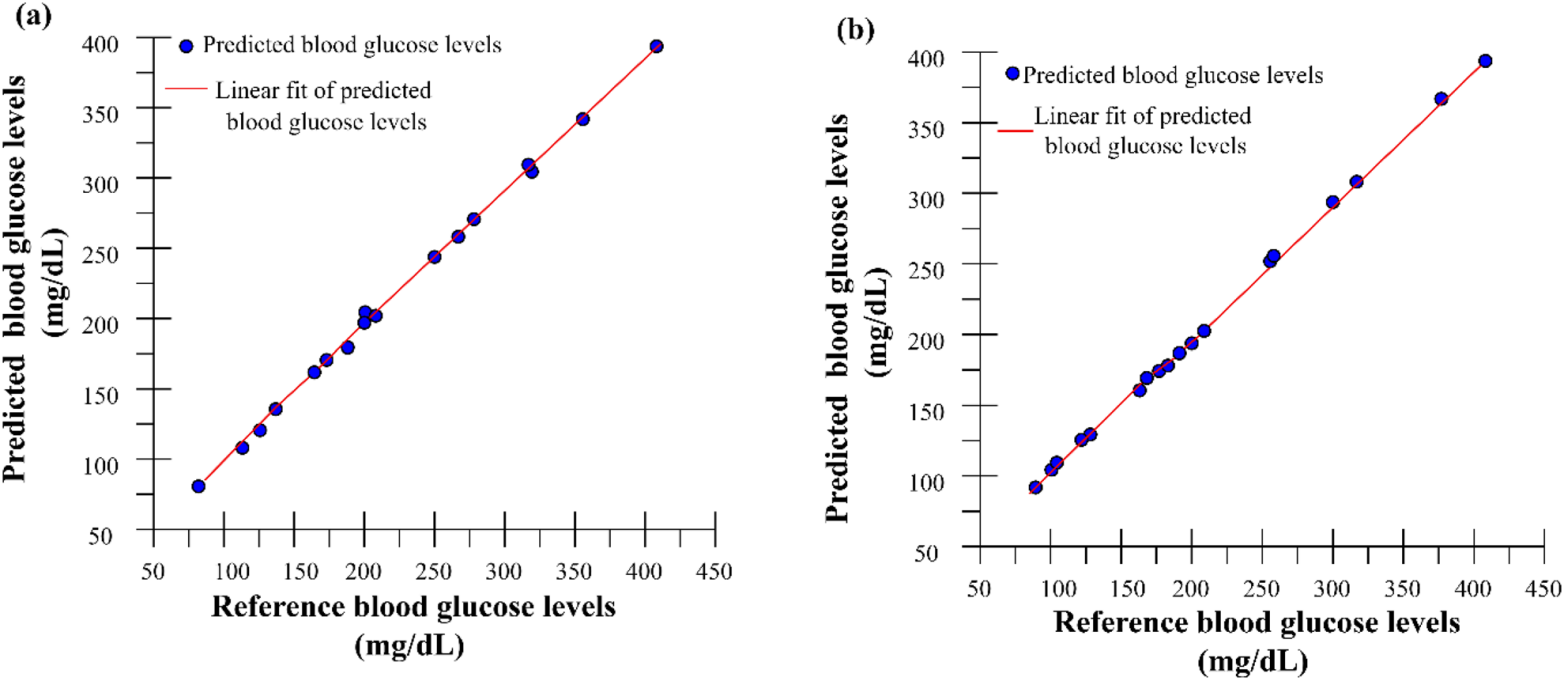
Validation of reference and predicted blood glucose levels niGLUC-2.0v: (**a**) Finger sensor; (**b**) Wrist sensor.

The validation of the proposed model, performance metrics, and visualizations through graphs are presented. A plot of the reference and predicted blood glucose levels on the proposed data analytic framework for the finger sensor is depicted in Fig. 7. The performance of niGLUC-2.0v is shown in Fig. 7a for the finger and Fig. 7b for the wrist sensor prototype. It can be inferred that the data points were nearest to the trend line, defining the correlation between the reference and predicted blood glucose levels. The X-axis represents the reference blood glucose levels, and the Y-axis represents the predicted blood glucose levels in mmol. The red line in the graph represents the prediction by RR. It can be inferred that the prediction and reference mmol values were closer, thus determining the good prediction accuracy in prediction.

The niGLUC-2.0v hardware was validated by performing error analysis. An error analysis was performed by evaluating the MAE. The model was tested using a new dataset, as presented in Tables 10 and 11. It can be inferred from Table 10 that the maximum error obtained was 1.92 mg/dL, and the minimum error obtained was **− **2.47 mg/dL for the finger sensor. The MAE obtained from 20 finger sensor data measurements was 0.15. Similarly, from Table 11, the maximum error obtained was 2.63 mg/dL, and the minimum error obtained was **− **0.02 mg/dL for the wrist sensor. The MAE obtained from the 20 wrist sensor data measurements was 0.068. It can be inferred that the training and testing MAE are the same for the finger and wrist sensor prototypes. The data analytic framework in niGLUC-2.0v has improved the performance of the model on the finger sensor with an accuracy of 99.02%, with MAE of 0.15 and MSE of 0.22 whereas, on the wrist sensor, the accuracy obtained was 99.96% with MAE of 0.06 and MSE of 0.006. The accuracy of both devices was within the clinically acceptable range. Therefore, the device developed is suitable for medical applications.

**Table 10:** Validation of proposed data analytic framework on finger sensor.

Reference BG (mmol)	Predicted BG (mmol)	Reference BG (mg/dL)	Predicted BG (mg/dL)	Error $(BG_{pred} - BG_{ref} )$ (mg/dL)
17.02	17.11	306.41	307.99	1.58
13.05	13.16	235.07	237.00	1.92
22.27	22.14	401.00	398.52	**− **2.47
10.69	10.56	192.49	190.09	**− **2.40
5.95	6.03	107.20	108.65	1.45

mmol: millimolecule; mg/dL: milligram/decilitre; BG: blood glucose; $BG_{pred}$: predicted blood glucose; $BG_{ref}$*:* reference blood glucose.

**Table 11:** Validation of proposed data analytic framework on wrist sensor.

Reference BG. (mmol)	Predicted BG. (mmol)	Reference BG. (mg/dL)	Predicted BG. (mg/dL)	Error $(BG_{pred} BG_{ref} )$(mg/dL)
22.27	22.28	401.00	401.14	0.14
14.01	14.16	252.33	254.97	2.63
5.36	5.47	96.49	98.57	2.07
10.09	10.18	181.66	183.24	1.57
19.22	19.22	345.99	345.97	**− **0.02

mmol: millimolecule; mg/dL: milligram/decilitre; BG: blood glucose; $BG_{pred}$:blood glucose predicted; $BG_{ref}$*:* blood glucose reference value.

### Bland–Altman analysis

**Figure 8:**
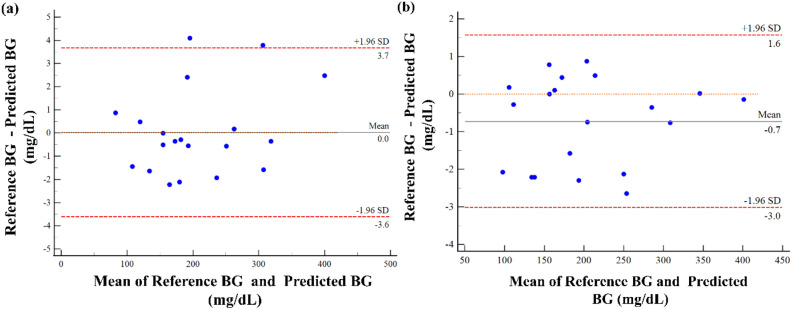
Bland Altman plot between reference and predicted blood glucose levels. (**a**) Finger sensor (**b**) Wrist sensor.

Bland–Altman analysis was used to analyze the difference between the predicted blood glucose levels and reference blood glucose levels. The limits of agreement (LOA) were at ± 1.96 Standard Deviations (SD) from the mean difference^77^. The Bland–Altman plot is illustrated in Fig. 8. The X-axis represents the mean of the reference and predicted blood glucose levels, whereas the Y-axis represents the difference between the reference and predicted blood glucose levels. It can be observed from Fig. 8a for the finger sensor, the mean difference/bias of blood glucose level was at 0.035 and 95% confidence interval between the upper and lower limits of agreement between + 3.7 and **− **3.6. Similarly, for the wrist sensor, as depicted in Fig. 8b, the mean difference/bias of blood glucose level was found at **− **0.7 and the 95% confidence interval lying between an upper and lower LOA was between + 1.6 and **− **3.0, indicating a strong correlation between the reference and predicted blood glucose levels.

### Clarke error grid analysis

**Figure 9:**
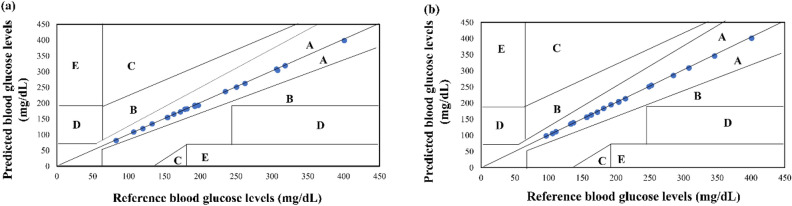
Clarke error grid analysis between reference and predicted blood glucose levels. (**a**) Finger sensor (**b**) wrist sensor.

CEG is an essential tool for evaluating the clinical accuracy of glucose monitoring devices^78^. Analysis was performed between the reference and predicted blood glucose levels. It can be observed from Fig. 9a and b that all the values fall under zone A of the grid which implies a high clinical significance of the sensor for its usage in the medical field for effective diabetic management.

### Statistical analysis

The data is subjected to statistical analysis where a paired t-test is carried out as the measurements were taken for the same subjects, i.e., between the reference and predicted blood glucose levels^79^. IBM SPSS software was used in the proposed work, where a null hypothesis and alternate hypothesis are presented in Eqs. (12) and (13) respectively. It was observed that the data were normal and the variances of differences were equal; therefore, no correction was needed. Paired t-test was applied to the dataset where *p* < 0.05 is set^50,64^. It can be observed from Table 12 that v0.001, and df = 100 were obtained with t-value = 0.59 for the finger and 0.56 for the wrist sensor leading to the acceptance of $H_{0}$,where it was concluded that there was no difference between the reference and predicted blood glucose levels.

**Table 12:** Paired t-test between reference and predicted blood glucose levels for finger and wrist sensor.

Sensor site	p-value	df	t-value
Finger	< 0.001	100	0.59
Wrist	< 0.001	100	0.56

df: degrees of freedom;

Null hypothesis ($H_{0}$): There is no significant difference between reference and predicted blood glucose levels.

16 $$H_{reference} = H_{predicted}$$

Alternate hypothesis ($H_{1}$): There is a significant difference between the reference and predicted blood glucose levels.

17 $$H_{reference} \ne H_{predicted}$$

For finger sensor^78,79^,

18 $${\text{p}} = 0.0{5}$$

19 $$t_{critical} = {1}.{66}0$$

20 $$t_{calculated} = 0.59$$

As $t_{calculated }$ < $t_{critical}$, the $H_{0}$ is accepted.

Similarly for the wrist sensor^79,80^,

21 $${\text{p}} = 0.0{5}$$

22 $$t_{critical} = {1}.{66}0$$

23 $$t_{calculated} = 0.{56}$$

As $t_{calculated }$ < $t_{critical}$, the $H_{0}$ is accepted.

### Comparison of niGLUC-2.0v with existing literature

The performance of the proposed device, i.e., niGLUC-2.0v, is compared with previous non-invasive approaches in Table 13. The proposed device was found to have greater accuracy with an R2_SCORE of 99.02%, MAE of 0.15, and MSE of 0.22, whereas R2_SCORE of 99.96%, MAE of 0.06, and MSE of 0.006 were obtained for the wrist sensor. The proposed sensor had the highest detection limit of 80–488 mg/dL compared to other studies. The integration of AI and Data Science with NIR technology has advanced other studies by accurately predicting the of blood glucose levels. The results from ridge regression, linear regression plot, Bland–Altman analysis, and CEG depict the high performance of both finger and wrist sensors.

**Table 13:** Comparison of non-invasive approaches in NIR spectroscopy with niGLUC-2.0v.

References	Wavelength implemented	Measurement site	Performance metrics
Sun et al.^28^	940 nm	Hand	RMSE. = 21.06 mg/dL
			MARD = 7.31%
			Clarke-error grid = 96% in clinically acceptable zones of A and B
Srichan et al.^29^	850 nm	Finger	Accuracy = 97.8%
			Precision = 96.0%
			Sensitivity = 94.8%
			Specificity = 98.7%
			Prediction error = ± 15
Haque et al.^30^	850 nm,950 nm,1150 nm	Finger	i. Haemoglobin
			· Accuracy = 92.2%
			ii. Blood glucose
			· Accuracy = 90.02%
			iii. Creatinine
			Accuracy = 96.9%
Heise, H. M et al.^31^	1100 nm,1825 nm	Lip	Standard prediction error = 36.6 mg/dL
			MARD = 23%
Joshi, A. M et al.^32^	940 nm,	Finger	i. Capillary blood glucose
	1300 nm		Average error = 6.09%
			MARD = 6.07%
			ii. Serum blood glucose
			Average error = 4.88%
			· MARD = 4.86%
Rachim, V. P et al.^33^	950 nm,850 nm, 660 nm,	Wrist	*Rp = 0.86*
	530 nm		SPE = 6.16 mg/dL
Proposed work (niGLUC-2.0v)	940 nm, (900–1700) nm	Fingertip	MAE = 0.15
			MSE = 0.22
			R2_SCORE = 99.02%
			Clarke-error grid = 100% in clinically acceptable zones of A and B
		Wrist	MAE = 0.06
			MSE = 0.006
			R2_SCORE = 99.96%
			Clarke-error grid = 100% in clinically acceptable zones of A and B

RMSE: Root mean square deviation; MARD: mean absolute relative difference; $R_{p}$: average correlation coefficient; SPE: standard percentage error; MAE: mean absolute error; MSE: mean square error; R2_SCORE: coefficient of determination.

The data analytic framework proposed in the current study provided the best accuracy in under the presence of ambiguities when compared to the current literature, with 99.02% for in the finger and 99.96% for in the wrist sensor. The statistical analysis with *p* < 0.05 strengthens the significance on the achieved accuracy which is not achieved in other works. The 100% data points of blood glucose values falling under zone A of CEG proved to be the best results when compared to the existing literature. The key feature of the proposed device is its ability to perform accurately in the presence of ambient light, pressure, and skin color variation, which has not been addressed in any of the existing literature. The measurement from the wrist sensor is accurately equal to that from finger sensor, which is less explored in the literature. The technological advancements explored in the proposed work with the integration of AI and data science prove to be efficient, stable, reliable, and smart for enhanced sensing accuracy when compared to the existing literature. As the proposed device is compact and portable, it is user-friendly compared to other studies. The high cost of the device is an additional advantage. The limitations of the existing methods, i.e., accuracy, cost of the device, effect of skin color variation, ambient light, pressure on the sensor, reliability, and testing the device on diabetic patients, are overcome by niGLUC-2.0v, which replaces existing non-invasive devices. The implication of a high degree of prediction accuracy in the device is essential for making informed medical decisions, such as treatment titrations and customized treatment plans, taking preventive measures, monitoring other chronic diseases along with diabetes, and avoiding suboptimal diagnosis. Therefore, with the accuracy of niGLUC-2.0v, it can be employed in hospitals and personal care as a one-time purchase gadget for monitoring blood glucose frequently and accurately with a reduced risk of complications and better diabetic management under convenience.

## Conclusion

The NIR with a 940 nm emitter and 900–1700 nm detector has been proved to measure blood glucose levels non-invasively. Owing to its noninvasive properties, it has potential benefits, including low cost and one-time investment in the device. The data analytic framework was developed to predict blood glucose levels non-invasively, and was validated by invasively obtained blood glucose levels. These results suggest that niGLUC-2.0v has the potential to accurately predict blood glucose levels and may be beneficial for better diabetic management. The proposed work has certain strengths: (i) The model was developed by considering the effects of variations in skin color, ambient light, and pressure on the device. Care has been taken to avoid motion artifacts by obtaining the measurements in a stable state. (ii) In this study, many volunteers (101), including diabetic and non-diabetic volunteers of all ages (20–90 years), measured blood glucose values invasively and non-invasively. The developed sensor system was validated on diabetic and non-diabetic volunteers by random sampling, i.e., fasting, postprandial, and random testing. (iii) Ambiguities faced are handled in niGLUC-2.0v. The limitation of the proposed work is that the device can only be tested in the stable state of a volunteer. The device was portable and not wearable. Future work is aimed at creating a miniaturized wearable version of the proposed system, that can be tested in motion with good accuracy.

### Supplementary Information

Supplementary Information 1.

Supplementary Information 2.

## Data Availability

The datasets used and/or analyzed during the current study are available from the corresponding author upon reasonable request.

## References

[1] Zhang W, Du Y, Wang ML (2015). Non-invasive glucose monitoring using saliva nano-biosensor. Sensing Bio-Sensing Res..

[2] Joshi S, Bhatt VD, Wu H, Becherer M, Lugli P (2017). Flexible lactate and glucose sensors using electrolyte-gated carbon nanotube field effect transistor for non-invasive real-time monitoring. IEEE Sens. J..

[3] Hathout E (2005). Home use of the GlucoWatch g2 biographer in children with diabetes. PEDIATRICS.

[4] Christiansen MP, Klaff LJ (2018). A prospective multicenter evaluation of the accuracy of a novel implanted continuous glucose sensor: PRECISE II. Diabetes Technol. Therapeut..

[5] Bode B (2004). Alarms based on real-time sensor glucose values alert patients to hypo- and hyperglycemia: The guardian continuous monitoring system. Diabetes Technol. Therapeut..

[6] Principles and problems of blood glucose measurement. (n.d.). Acutecaretesting.org. https://acutecaretesting.org/en/articles/principles-and-problems-of-blood-glucose-measurement (2022).

[7] Kiani S, Rezaei P (2023). Microwave substrate integrated waveguide resonator sensor for non-invasive monitoring of blood glucose concentration: Low cost and painless tool for diabetics. Measurement.

[8] Kiani S, Rezaei P, Fakhr M (2023). Real-time measurement of liquid permittivity through label-free meandered microwave sensor. IETE J. Res..

[9] Juan CG (2021). Study of Q_u_-based resonant microwave sensors and design of 3-D-printed devices dedicated to glucose monitoring. IEEE Trans. Instrum. Meas..

[10] Piyush KM, Vijay ST (2023). A compact dual-band hybrid dielectric resonator antenna for blood glucose sensing and wireless communication. Opt. Quant. Electron..

[11] Kiani S, Rezaei P, Fakhr M (2021). Dual-frequency microwave resonant sensor to detect noninvasive glucose-level changes through the fingertip. IEEE Trans. Instrum. Meas..

[12] Mohammadi P, Mohammadi A, Demir S, Kara A (2021). Compact size, and highly sensitive, microwave sensor for non-invasive measurement of blood glucose level. IEEE Sensors J..

[13] Baghelani M, Abbasi Z, Daneshmand M, Light PE (2020). Non-invasive continuous-time glucose monitoring system using a chipless printable sensor based on split ring microwave resonators. Sci. Rep..

[14] Kazemi N, Abdolrazzaghi M, Light PE (2023). Petr MusilekIn–human testing of a non-invasive continuous low–energy microwave glucose sensor with advanced machine learning capabilities. Biosensors Bioelectron..

[15] Wu W, Xiao X, Wang Z, Sun J, Zhang X (2024). Highly sensitive blood glucose monitoring sensor with adjustable resonant frequency based on MP-CSRR. Sensors Actuators A Phys..

[16] Villena Gonzales W, Mobashsher A, Abbosh A (2019). The Progress of glucose monitoring—a review of invasive to minimally and non-invasive techniques Devices and Sensors. Sensors.

[17] Wu J, Liu Y, Yin H, Guo M (2023). A new generation of sensors for non-invasive blood glucose monitoring. Am. J. Transl. Res..

[18] Monograph: A guide to near-infrared spectroscopic analysis of industrial manufacturing processes (n.d.) https://www.metrohm.com/en_in/products/8/1085/81085026.html

[19] CoG - Hybrid Glucometer|Cnoga Digital Care (n.d.) Cnoga Care. https://www.cnogacare.co/cog-hybrid-glucometer

[20] Pfützner A (2019). System accuracy assessment of a combined invasive and noninvasive glucometer. J. Diabetes Sci. Technol..

[21] Heloextense. (n.d.). WGN. Retrieved March 2, 2024, from https://website.worldgn.com/heloextense/

[22] Litvinova O (2023). Patent analysis of digital sensors for continuous glucose monitoring. Front. Public Health.

[23] Hadar E, Chen R, Toledano Y, Tenenbaum-Gavish K, Atzmon Y, Hod M (2019). Noninvasive, continuous, real-time glucose measurements compared to reference laboratory venous plasma glucose values. J. Maternal-Fetal Neonatal Med..

[24] Padmavilochanan D, Pathinarupothi RK, Menon KAU, Kumar H, Guntha R, Ramesh MV, Rangan PV (2023). Personalized diabetes monitoring platform leveraging IoMT and AI for non-invasive estimation. Smart Health.

[25] Mosaddequr K, Rahman T (2023). A novel multipurpose device for dataset creation and on-device immediate estimation of blood glucose level from reflection ppg. Heliyon.

[26] Argüello-Prada EJ, Bolaños SM (2023). On the role of perfusion index for estimating blood glucose levels with ultrasound-assisted and conventional finger photoplethysmography in the near-infrared wavelength range. Biomed. Signal Process. Control.

[27] Darwich MA, Shahen A, Daoud A, Lahia A, Diab J, Ismaiel E (2023). Non-invasive IR-based measurement of human blood glucose. Eng. Proc..

[28] Sun Y (2023). Random forest analysis of combined millimeter-wave and near-infrared sensing for noninvasive glucose detection. IEEE Sensors J..

[29] Srichan C, Srichan W, Danvirutai P, Ritsongmuang C, Sharma A, Anutrakulchai S (2022). Non-invasively accuracy enhanced blood glucose sensor using shallow dense neural networks with NIR monitoring and medical features. Sci. Rep..

[30] Haque MdR, Raju SMTU, Golap M-U, Hashem MMA (2021). A Novel technique for non-invasive measurement of human blood component levels from fingertip video using DNN based models. IEEE Access.

[31] Heise HM, Delbeck S, Marbach R (2021). Noninvasive monitoring of glucose using near-infrared reflection spectroscopy of skin—constraints and effective novel strategy in multivariate calibration. Biosensors.

[32] Joshi AM, Jain P, Mohanty SP, Agrawal N (2020). iGLU 20: a new wearable for accurate non-invasive continuous serum glucose measurement in IoMT framework. IEEE Trans. Consumer Electron..

[33] Rachim VP, Chung W-Y (2019). Wearable-band type visible-near infrared optical biosensor for non-invasive blood glucose monitoring. Sensors Actuat. B Chem..

[34] Yadav J, Rani A, Singh V, Murari BM (2015). Prospects and limitations of non-invasive blood glucose monitoring using near-infrared spectroscopy. Biomed. Signal Process. Control.

[35] Burmeister JJ, Arnold MA, Small GW (2000). Noninvasive blood glucose measurements by near-infrared transmission spectroscopy across human tongues. Diabetes Technol. Therapeut..

[36] Lee SH, Cho YC, Bin Choy Y (2019). Noninvasive self-diagnostic device for tear collection and glucose measurement. Sci. Rep..

[37] Li, T., Bai, D., Prioleau, T., Bui, N., Vu, T., & Zhou, X. Noninvasive glucose monitoring using polarized light. in *SenSys ’20: Proceedings of the 18th Conference on Embedded Networked Sensor Systems*. 10.1145/3384419.343072 (2020).

[38] Fu Y, Huang M, Chen X (2021). Fingertip capillary dynamic near infrared spectrum (DNIRS) measurement combined with multivariate linear modification algorithm for noninvasive blood glucose monitoring. Vib. Spectrosc..

[39] Nakayama T, Kohdera U, Fujino M, Tanaka T, Yatabe K, Hashiguchi T, Sato T, Kino M (2016). Appropriate needle lengths determined using ultrasonic echograms for intramuscular injections in Japanese infants. Open J. Pediat..

[40] JahangiriNoudeh Y, Hadaegh F, Vatankhah N, Momenan AA, Saadat N, Khalili D, Azizi F (2013). Wrist circumference as a novel predictor of diabetes and prediabetes: results of cross-sectional and 88-year follow-up studies. J. Clin. Endocrinol. Metab..

[41] Jones S, Chiesa ST, Chaturvedi N, Hughes AD (2016). Recent developments in near-infrared spectroscopy (NIRS) for the assessment of local skeletal muscle microvascular function and capacity to utilise oxygen. Artery Res..

[42] Andersen J-H (2019). Bioimpedance and NIR for non-invasive assessment of blood glucose. J. Electr. Bioimpedance.

[43] Kaysir MR, Song J, Rassel S, Aloraynan A, Ban D (2023). Progress and perspectives of mid-infrared photoacoustic spectroscopy for non-invasive glucose detection. Biosensors.

[44] Ahmed I, Jiang N, Shao X, Elsherif M, Alam F, Salih A, Butt H, Yetisen AK (2022). Recent advances in optical sensors for continuous glucose monitoring. Sensors Diagnost..

[45] Hina A, Saadeh W (2022). Noninvasive blood glucose monitoring systems using near-infrared technology—a review. Sensors.

[46] Maier JS, Walker SA, Fantini S, Franceschini MA, Gratton E (1994). Possible correlation between blood glucose concentration and the reduced scattering coefficient of tissues in the near infrared. Opt. Lett..

[47] Braverman IM (2000). The cutaneous microcirculation. J. Investig. Dermatol. Symp. Proc..

[48] Finlayson L, Barnard IRM, McMillan L, Ibbotson SH, Brown CTA, Eadie E, Wood K (2021). Depth penetration of light into skin as a function of wavelength from 200 to 1000 nm. Photochem. Photobiol..

[49] Laha S, Rajput A, Laha SS, Jadhav R (2022). A concise and systematic review on non-invasive glucose monitoring for potential diabetes management. Biosensors.

[50] Campbell JD, Holder-Pearson L, Pretty CG, Benton C, Knopp J, Chase JG (2020). Development of a discrete spectrometric NIR reflectance glucometer. IFAC-PapersOnLine.

[51] Koster, P. Near infrared light penetration in human tissue: An analysis of tissue structure and heterogeneities. Master’s Theses (2009). https://epublications.marquette.edu/theses_open/739/ (2022).

[52] Konig K (2000). Multiphoton microscopy in life sciences. J. Microsc..

[53] Tenhunen J, Kopola H, Myllylä R (1998). Non-invasive glucose measurement based on selective near infrared absorption; requirements on instrumentation and spectral range. Measurement.

[54] Anderson RR, Parrish JA (1981). The optics of human skin. J. Investig. Dermatol..

[55] Al-Fahoum AS, Al-Zaben A, Seafan W (2015). A multiple signal classification approach for photoplethysmography signals in healthy and athletic subjects. Int. J. Biomed. Eng. Technol..

[56] Goh H (2017). Types of circuit breaker and its application in substation protection. Indonesian J. Electr. Eng. Comput. Sci..

[57] Mayuri A. *et al*. Study and analysis of different types of circuit breaker. Int. J. Adv. Res. Sci. Commun. Technol. (2022) 10.48175/ijarsct-3041

[58] International Commission on Non-Ionizing Radiation Protection ICNIRP Guidelines on Limits of Exposure to Incoherent Visible and Infrared Radiation. (N.D.). https://www.icnirp.org/cms/upload/publications/ICNIRPVisible_Infrared2013.pdf 10.1097/HP.0b013e318289a611 35606999

[59] Book Lab Tests at Home from Apollo Diagnostics, Pathology Labs near me. (n.d.). www.apollo247.com. https://www.apollo247.com/lab-tests

[60] Accu-Chek Active Blood Glucose Glucometer Kit With Vial Of 10 Strips, 10 Lancets And A Lancing Device Free For Accurate Blood Sugar Testing: Amazon.in: Health & Personal Care. (n.d.). https://www.amazon.in/Accu-Chek-Active-Glucose-strips-Multicolor/dp/B01GO0HBF6

[61] etheme.com. (n.d.). DexCom G6 sensors & G6 transmitter combo. Diabetic Warehouse. https://www.diabeticwarehouse.org/products/dexcom-g6-sensors-g6-transmitter-combo

[62] Insurance Coverage for Eversense® E3 CGM System|Ascensia Diabetes Care. (n.d.). https://www.ascensiadiabetes.com/eversense/coverage/insurance-and-cost/

[63] Yadav, J., Rani, A., Singh, V., & Murari, B.M. Near-infrared LED based non-invasive blood glucose sensor. In *2014 International Conference on Signal Processing and Integrated Networks (SPIN)*, 591–594. - References - Scientific Research Publishing. (n.d.). www.scirp.org. Retrieved May 13, 2023, from https://www.scirp.org/(S(351jmbntvnsjt1aadkposzje))/reference/ReferencesPapers.aspx?ReferenceID=1532659 (2014)

[64] Li A, Fan M, Qin G, Xu Y, Wang H (2021). Comparative analysis of machine learning algorithms in automatic identification and extraction of water boundaries. Appl. Sci..

[65] Çakıt E, Dağdeviren M (2023). Comparative analysis of machine learning algorithms for predicting standard time in a manufacturing environment. Artif. Intell. Eng. Des. Anal. Manuf..

[66] Raza, A., Faiz-Ur-Rehman, B.M., & Rauf, M. Comparative analysis of machine learning algorithms for fake review detection. *Int. J. Comput. Intell. Control* **13**(1) (2021).

[67] Vishnepolsky B (2022). Comparative analysis of machine learning algorithms on the microbial strain-specific AMP prediction. Brief. Bioinformat..

[68] Lampe L (2022). Comparative analysis of machine learning algorithms for multi-syndrome classification of neurodegenerative syndromes. Alzheimer’s Res. Therapy.

[69] Al-Fahoum AS, Abu O, Hussam A (2023). Identification of coronary artery diseases using photoplethysmography signals and practical feature selection process. Bioengineering.

[70] Al Fahoum, A., Al Omari, A., Al Omari, G., & Ala'a Zyout. (n.d.). PPG signal-based classification of blood pressure stages using wavelet transformation and pre-trained deep learning models. 10.22489/CinC.2023.360 (2023).

[71] Sarker IH (2021). Machine learning: algorithms, real-world applications and research directions. SN Comput. Sci..

[72] Shi R, Leng X, Wu Y, Zhu S, Cai X, Lu X (2023). Machine learning regression algorithms to predict short-term efficacy after anti-VEGF treatment in diabetic macular edema based on real-world data. Sci. Rep..

[73] Piri, M. Review of regression algorithms. (2023).

[74] Shirai S, Kudo M, Nakamura A (2009). Comparison of bagging and boosting algorithms on sample and feature weighting. Lect. Notes Comput. Sci..

[75] MindSea. *Patient demographics: How they can improve healthcare*. MindSea Development. https://mindsea.com/patient-demographics (2020).

[76] Slavin W (1963). Stray light in ultraviolet, visible, and near-infrared spectrophotometry. Anal. Chem..

[77] Giavarina D (2015). Understanding bland Altman analysis. Biochem. Med. (Zagreb).

[78] Clarke WL, Cox D, Gonder-Frederick LA, Carter W, Pohl SL (1987). Evaluating clinical accuracy of systems for self-monitoring of blood glucose. Diabetes Care.

[79] Ross A, Willson VL (2017). Paired samples T-Test. Basic Adv. Stat. Tests.

[80] NIST.1.3.6.7.2. Critical Values of the Student’s-t Distribution. Nist.gov. https://www.itl.nist.gov/div898/handbook/eda/section3/eda3672.htm (2020).

