# Supplementary material for: Development of sensor system and data analytic framework for non-invasive blood glucose prediction
Source: Sci Rep. 2024 Apr 22;14:9206. doi: 10.1038/s41598-024-59744-7 (PMC11035575; doi:10.1038/s41598-024-59744-7)
Supplement: Supplementary file 1 — Supplementary Information 1. [file 41598_2024_59744_MOESM1_ESM.docx]

**I1**

This section presents the exceptional studies carried out in the areas of the thermal, electric/electromagnetic, and optical methods for non-invasive glucose monitoring. Metabolic Heat Conformation (MHC) and Thermal Emission Spectroscopy (TES) are implemented to measure glucose concentration with metabolic heat produced by the body. The drawback of thermal method, i.e., its susceptibility to sweat, motion, and temperature, remains a major concern for non-invasive blood glucose measurements [1]. Bioimpedance spectroscopy (BS), electromagnetic sensing (EMS), millimeter wave (mmW), and microwave (µm) are different areas of electric and electromagnetic methods for measuring blood glucose non-invasively. Similar to the thermal method, the BS and EMS methods are sensitive to temperature, motion, sweat, and water [1]. In recent times, research work has been carried out on millimeter wave, where a meta material inspired resonator with five split rings was implemented at 24.9 GHz and a Frequency Modulated Continuous Wave (FMCW) was implemented between 50 and 67 GHz using a radar sensor [2-3]. The major limitation of this approach is its susceptibility to background noise, biological differences in blood, breathing, sweating level, cardiac activity, poor selectivity, and non-portability [1,4]. Moreover, the work was not executed on real time blood samples, which may have led to a bias in the observation.


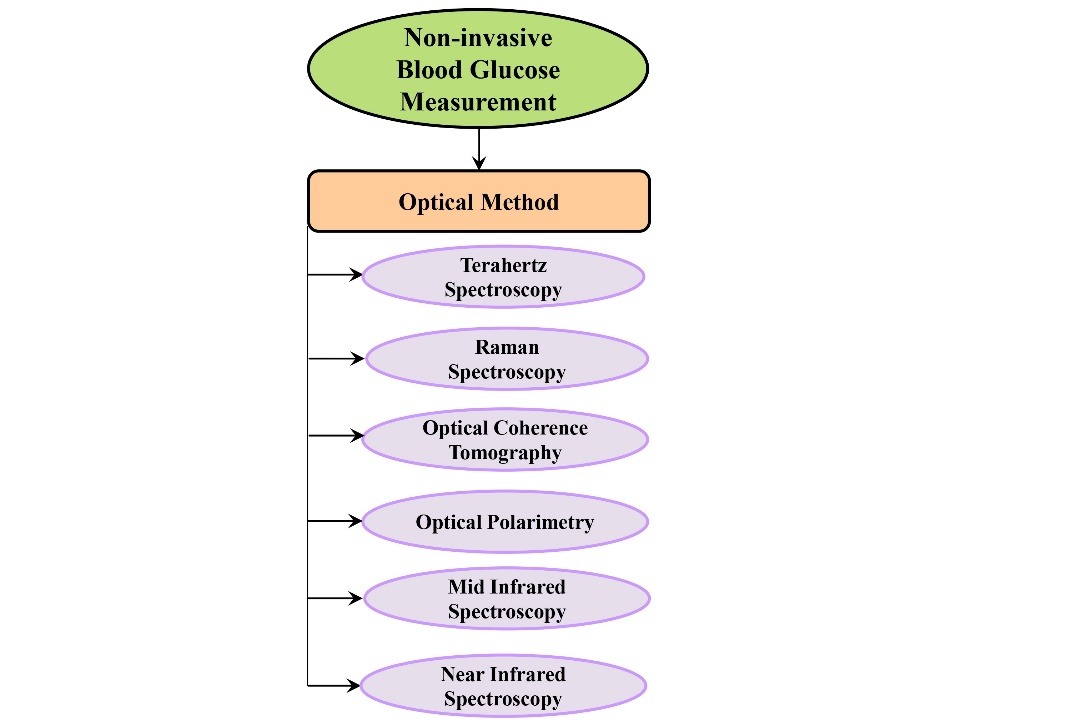


Figure 1. Methods of Non-invasive blood glucose measurement in Optical Method

The non-invasive glucose monitoring solution is pain-free, stress-free, and requires continuous glucose monitoring. Recently, there has been significant interest in developing non-invasive glucose monitoring devices using saliva, skin, fingertip, earlobe, urine, wrist, lip, and aqueous solution [15-5]. These methodologies have the drawback of being non-portable and non-wearable, making it impossible to continuously measure real-time blood glucose. Excellent research has been carried out in optical-spectroscopy-based non-invasive glucose monitoring, as depicted in Fig 1. THz spectroscopy at (100-300 GHz) was implemented to measure blood glucose from blood vessels between the thumb-index area. The results show the device has 2 dB sensitivity for 15 mg/dL change in blood glucose concentration with a prediction of ±5%. The blood glucose concentration is measured through aqueous glucose samples in the range of 70 to 145 mg/dL [6]. Raman spectroscopy was proposed in a study to measure blood glucose levels from nail fold. Principal component analysis (PCA) and back propagation artificial neural networks (BP-ANN) was implemented to predict the blood glucose levels. A root mean square error of prediction (RMSEP) of 0.27 mmol/L and an R^2^ of 0.98 were obtained, which depicts linearity between predicted blood glucose levels and reference blood glucose levels [7]. In another study, optical coherence tomography (OCT) and a genetic algorithm were implemented to determine blood glucose levels from the finger by retrieving the optical rotation angle and depolarization index. Glucose concentration is measured in an aqueous solution varying from 0 to 4000 mg/dL, with an R^2^ of 0.91 and an average standard deviation of 0.125 for optical rotation angle and a depolarization index with an R^2^ of 0.59 and an average standard deviation of 0.027 [8]. In a study of optical polarimetry, a blood glucose monitoring system is developed with a size of 17cm×10cm×5cm and is low-cost, i.e., $250. The absolute relative difference (ARD) of 10% with an accuracy of 89% and a Pearson correlation of r=0.91 and p=1.6× 10^-143^ is achieved [9]. A mid-infrared spectroscopy-based wrist imaging spectrometer is developed with dimensions of 105 mm × 90 mm × 50 mm and a weight of 1.25 kg. Glucose peaks are observed at 9250 nm and 9650 nm. A correlation of R=0.86 is obtained between glucose concentration and emittance [10]. However, few experimental studies that there is a strong absorption of water in the terahertz region ,which makes it difficult to obtain insightful data [11-14].Similarly, Raman spectroscopy is prone to interference from hemoglobin, hemocrit, skin thickness, melanin, low signal-to-noise ratio (SNR) and turbidity [15-18].Despite of OCT having a potential of high SNR, the temperature changes in the skin and susceptibility to motion, tissue permittivity, and conductivity limit the implementation of OCT in detecting blood glucose [18-22].The presence of weak optical signals beneath the skin, the temperature changes in the skin, and the susceptibility to motion, unable to be detected [9,18,20,23]. Shallow penetration depth of 10 µm, strong water absorption, and interreference of hemoglobin are found to interfere while detecting blood glucose [18,24-26]. NIR spectroscopy is weak enough to detect hemoglobin and water, which makes it suitable for detecting glucose molecules. In NIR, the intensity of the signal is directly proportional to the glucose molecule, with a good penetration of 6 mm even in the presence of glass or plastic container [18, 27-30]. The cost of materials for the NIR emitter and detector is low and compact when compared with other optical alternatives. The scattering of light is high in the NIR with heterogeneous glucose molecules. By accounting for the scattering and heterogeneity of glucose molecules, a non-invasive, low-cost, small device can be built.

**I2**

Glucowatch G2 Biographer works on the principle of iontophoresis. It was discontinued due to allergic skin reactions and its inability to detect hypoglycemia [31-32]. A similar drawback is found in OrSense NBM-200G based on Occlusion spectroscopy of non-invasive monitoring [33-34]. Glucotrack by Integrity Applications is a hybrid model based on electromagnetic, ultrasonic, and thermal techniques. Blood glucose levels are detected from the earlobe by attaching a clip to the ear, making it non-portable [35-36]. Sugarbeat, released by Nemaura Medical, requires a one-time finger prick for calibration. Based on reverse iontophoresis, blood glucose levels are monitored from the legs, arms, and abdomen with a Mean Absolute Relative Difference (MARD) of 12.4% [37].

**References**

1. Villena Gonzales, W., Mobashsher, A., & Abbosh, A. The Progress of Glucose Monitoring—A Review of Invasive to Minimally and Non-Invasive Techniques, Devices and Sensors. Sensors, **19**(4), 800. <https://doi.org/10.3390/s19040800> (2019).
2. Qureshi S.A. *et al.* Glucose level detection using millimetre-wave metamaterial-inspired resonator. PLOS ONE, **17**(6), e0269060–e0269060. <https://doi.org/10.1371/journal.pone.0269060> (2022).
3. Omer, A. E., Safavi-Naeini, S., Hughson, R., & Shaker, G. Blood Glucose Level Monitoring Using an FMCW Millimeter-Wave Radar Sensor. Remote Sensing, **12**(3), 385. <https://doi.org/10.3390/rs12030385> (2020).
4. Tang, L., Chang, S. J., Chen, C. J., & Liu, J. T. Non-Invasive Blood Glucose Monitoring Technology: A Review. *Sensors (Basel, Switzerland)*, **20**(23), 6925. <https://doi.org/10.3390/s20236925> (2020).
5. Kalaivani V,Devika E,Arulladakanthan R & Santoshini Arulvallal, S. Kalaivani.Designing and implementation of noninvasive blood glucose and hemoglobin detection using NIR. *International Journal of Innovative Technology and Exploring Engineering*, **9**, no. 12, pp. 229–232, 2020.
6. P. Kaurav, S. K. Koul, and A. Basu.Non-invasive glucose measurement using sub-terahertz sensor, time domain processing, and neural network.*IEEE Sens. J.*, **21**, no. 18, pp. 20002–20009, 2021.
7. S. I. Gusev, M. A. Borovkova, M. A. Strepitov, and M. K. Khodzitsky.Blood optical properties at various glucose level values in THz frquency range. in *Clinical and Biomedical Spectroscopy and Imaging IV*, (2015).
8. Chen, T. L., Lo, Y. L., Liao, C. C., & Phan, Q. H. Noninvasive measurement of glucose concentration on human fingertip by optical coherence tomography. *Journal of biomedical optics*, **23**(4), 1–9. <https://doi.org/10.1117/1.JBO.23.4.04700111> (2018).
9. P. Narkhede, S. Dhalwar, and B. Karthikeyan.NIR based non-invasive blood glucose measurement. *Indian J. Sci. Technol.*, **9**, no. 41, pp. 1–7, (2016).
10. P. Jain, A. M. Joshi, and S. P. Mohanty.IGLU: An intelligent device for accurate noninvasive blood glucose-level monitoring in smart healthcare. IEEE consum. electron. mag., **9**, no. 1, pp. 35–42, (2020).
11. M. R. Haque, S. M. T. U. Raju, M. A.-U. Golap, and M. M. A. Hashem.A novel technique for non-invasive measurement of human blood component levels from fingertip video using DNN based models.*IEEE Access*, **9**, pp. 19025–19042, (2021).
12. Kalaivani V,Devika E,Arulladakanthan R & Santoshini Arulvallal, S. Kalaivani.Designing and implementation of non invasive blood glucose and hemoglobin detection using NIR.*International Journal of Innovative Technology and Exploring Engineering*, **9**, no. 12, pp. 229–232, (2020).
13. S. Parama, A. Asm Shamsul, and M. I. Abu Shahadat.A feasibility study of non-invasive blood glucose level detection using near-infrared optical spectroscopy.*Banglad. J. Med. Phys.*, **14**, no. 1, pp. 1–13, 2021.
14. CoG - Hybrid Glucometer | Cnoga Digital Care. (n.d.). Cnoga Care. <https://www.cnogacare.co/cog-hybrid-glucomete>
15. NBM 200 - Orsense. (n.d.). [Www.orsense.com](http://Www.orsense.com). <http://www.orsense.com/product.php?ID=4>.
16. Amir, O. *et al*.Continuous noninvasive glucose monitoring technology based on ‘occlusion spectroscopy. *J. Diabetes Sci. Technol.*,**1**, no. 4, pp. 463–469.(2007).
17. Inc, G. (2021, November 29). Integrity Applications Announces Name Change to GlucoTrack, Inc. GlobeNewswire News Room. <https://www.globenewswire.com/en/news-release/2021/11/29/2342184/0/en/Integrity-Applications-Announces-Name-Change-to-GlucoTrack-Inc.html>
18. T. Lin, Y. Mayzel, and K. Bahartan.The accuracy of a non-invasive glucose monitoring device does not depend on clinical characteristics of people with type 2 diabetes mellitus. *J. Drug Assess.*, **7**, no. 1, pp. 1–7, (2018).
19. Nemaura Medical. (n.d.). Nemaura Medical. <https://nemauramedical.com>
20. Inc, G. *Integrity Applications Announces Name Change to GlucoTrack, Inc.* GlobeNewswire News Room. <https://www.globenewswire.com/en/news-release/2021/11/29/2342184/0/en/Integrity-Applications-Announces-Name-Change-to-GlucoTrack-Inc.html>
21. American Diabetes Association. (2023). Diagnosis | ADA. Diabetes.org. <https://diabetes.org/about-diabetes/diagnosis>
22. Mayo Clinic. (2017). *Prediabetes - Diagnosis and treatment - Mayo Clinic*. Mayoclinic.org. <https://www.mayoclinic.org/diseases-conditions/prediabetes/diagnosis-treatment/drc-20355284>
23. Book Lab Tests at Home from Apollo Diagnostics, Pathology Labs near me. (n.d.). Www.apollo247.com. <https://www.apollo247.com/lab-test>.
24. Accu-Chek Active Blood Glucose Glucometer Kit With Vial Of 10 Strips, 10 Lancets And A Lancing Device Free For Accurate Blood Sugar Testing : Amazon.in: Health & Personal Care. (n.d.). Www.amazon.in. <https://www.amazon.in/Accu-Chek-Active-Glucose-strips-Multicolor/dp/B01GO0HBF6>
25. etheme.com. (n.d.). DexCom G6 Sensors & G6 Transmitter Combo. Diabetic Warehouse. <https://www.diabeticwarehouse.org/products/dexcom-g6-sensors-g6-transmitter-combo>
26. *Insurance Coverage for Eversense® E3 CGM System | Ascensia Diabetes Care*. (n.d.). Www.ascensiadiabetes.com. <https://www.ascensiadiabetes.com/eversense/coverage/insurance-and-cost/>
27. Beć, K. B., Grabska, J., & Huck, C. W. (2020). Near-Infrared Spectroscopy in Bio-Applications. Molecules, 25(12). <https://doi.org/10.3390/molecules25122948>
28. Agelet, L. E., & Hurburgh, C. R. (2010). A Tutorial on Near Infrared Spectroscopy and Its Calibration. *Critical Reviews in Analytical Chemistry*, *40*(4), 246–260. <https://doi.org/10.1080/10408347.2010.515468>
29. Uwadaira, Y., Adachi, N., Ikehata, A., & Kawano, S. (2010). Factors Affecting the Accuracy of Non-Invasive Blood Glucose Measurement by Short-Wavelength near Infrared Spectroscopy in the Determination of the Glycaemic Index of Foods. Journal of near Infrared Spectroscopy, 18(5), 291–300. <https://doi.org/10.1255/jnirs.895>
30. Gao, Y. *et al*. A high sensitive glucose sensor based on Ag nanodendrites/Cu mesh substrate via surface-enhanced Raman spectroscopy and electrochemical analysis. Journal of Alloys and Compounds, 863, 158758. <https://doi.org/10.1016/j.jallcom.2021.158758> (2021).
31. CoG - Hybrid Glucometer | Cnoga Digital Care. (n.d.). Cnoga Care. https://www.cnogacare.co/cog-hybrid-glucometerNBM 200 - Orsense. (n.d.). Www.orsense.com. Retrieved May 13, 2023, from <http://www.orsense.com/product.php?ID=49>
32. NBM 200 - Orsense. (n.d.). Www.orsense.com. Retrieved May 13, 2023, from <https://www.orsense.com/product.php?ID=49>
33. Lin, T., Mayzel, Y., & Bahartan, K. The accuracy of a non-invasive glucose monitoring device does not depend on clinical characteristics of people with type 2 diabetes mellitus. Journal of Drug Assessment, 7(1), 1–7. https://doi.org/10.1080/21556660.2018.1423987 (2018).
34. Nemaura Medical. (n.d.). Nemaura Medical <https://nemauramedical.com/>
35. https://www.facebook.com/WebMD. Prediabetes (Borderline Diabetes). WebMD; WebMD. https://www.webmd.com/diabetes/what-is-prediabetes (2003).
36. American Diabetes Association. Diagnosis | ADA. Diabetes.org. https://diabetes.org/diabetes/a1c/diagnosis (2022).
37. Sridevi, P., Arefin, A. S., & Md Ibrahim, A. S. A Feasibility Study of Non-invasive Blood Glucose Level Detection Using Near-Infrared Optical Spectroscopy. Bangladesh Journal of Medical Physics, 14(1), 1–13. https://doi.org/10.3329/bjmp.v14i1.57313 (2021).
