# Supplementary material for: Development of sensor system and data analytic framework for non-invasive blood glucose prediction
Source: Sci Rep. 2024 Apr 22;14:9206. doi: 10.1038/s41598-024-59744-7 (PMC11035575; doi:10.1038/s41598-024-59744-7)
Supplement: Supplementary file 2 — Supplementary Information 2. [file 41598_2024_59744_MOESM2_ESM.docx]

**T1.** Developed prototypes in the field of non-invasive blood glucose monitoring

| Ref | Proposed work | Methodology applied | Results |
| --- | --- | --- | --- |
| Sun,Y *et.al* [30] | Development of a multi-sensor system with mm-wave and NIR for detecting blood glucose levels. | RF algorithm | - RMSE = 21.06mg/dL - MARD = 7.31% - Clarke-error grid = 96% in clinically acceptable zones of A and B. |
| Srichan, C *et.al* [31] | Monitoring blood glucose from mbNIR sensor and medical features. | SDNN | - Accuracy = 97.8% - Precision = 96.0% - Sensitivity= 94.8% - Specificity=98.7% - Prediction error=±15 |
| Haque, Md. R *et.al* [32] | Detection of hemoglobin, glucose, and creatinine. | Genetic algorithm and DNN. | 1. Haemoglobin  - Accuracy = 92.2%  1. Blood glucose  - Accuracy= 90.02%  1. Creatinine  - Accuracy = 96.9% |
| Heise, H. M *et.al* [33] | Blood glucose detection using transcutaneous reflection skin spectra | Multivariate calibration using SBC and Monte Carlo simulations. | - Standard prediction error = 36.6 mg/dL - MARD = 23% |
| Joshi, A. M *et.al* [34] | Development of blood glucose sensor system from capillary and serum blood glucose. | MPR | i.Capillary blood glucose   - Average error=6.09% - MARD=6.07%   ii.Serum blood glucose   - Average error=4.88% - MARD=4.86% |
| Rachim, V. P *et.al* [35] | Development of band-type wearable optical bio-sensor for blood glucose measurement. | DWT and WD. | - $R_{p}=$0.86 - SPE = 6.16 mg/dL |

NIR: Near infrared; RF: Random Forest; RMSE: Root mean square deviation; MARD: Mean Absolute Relative Difference; mbNIR: multiple photonic band Near Infrared; SDNN: Shallow Dense Neural Network; DNN: Deep Neural Network; SBC: Science based calibration; MPR: Multiple Polynomial Regression; DWT: Digital Wavelet Transform; WD: Wavelet Decomposition; $R_{p}$:Average correlation coefficient; SPE: Standard Percentage Error
